# Comprehensive characterization of the genes in AP2/ERF family and their involvement in salt–alkali stress response during *Nelumbo nucifera* seed germination

**DOI:** 10.3389/fpls.2026.1922760

**Published:** 2026-08-27

**Authors:** Rong Wang, Peiying Liu, Shuai Li, Song Sheng, Shuoli Zheng, Xiumei Yang, Wentao Huang, Qilong Liu, Zishan Shu, Siyao Wang, Donglv Shu

**Affiliations:** 1 Flower Research Institute, Hunan Botanical Garden, Changsha, China; 2 National & Local United Engineering Laboratory of Intergrative Utilization Technology of Eucommia Ulmoides, Jishou University, Jishou, China; 3 College of Landscape Architecture, Central South University of Forestry and Technology, Changsha, China; 4 College of Biology, Hunan University, Changsha, China; 5 College of Forestry, Central South University of Forestry and Technology, Changsha, China; 6 Yueyang Agricultural Science Research Institute, Yueyang, China; 7 Yongan Primary School Affiliated to Yantian District Institute of Education, Shenzhen, China

**Keywords:** AP2/ERF transcription factors, *Nelumbo nucifera*, salt–alkali stress, seed germination process, transcriptome analysis

## Abstract

*Nelumbo nucifera* Gaertn. is an economically and ecologically important aquatic plant, but its growth and productivity are severely constrained by soil salinization and alkalization. AP2/ERF transcription factors are key regulators of plant abiotic stress responses; however, their roles in salt–alkali tolerance in *N. nucifera* remain largely unclear. In this study, we performed a genome-wide identification and characterization of the AP2/ERF gene family in *N. nucifera*, followed by phylogenetic, structural, and physicochemical analyses. A total of 101 AP2/ERF genes were identified and classified into five subfamilies, showing both evolutionary conservation and species-specific divergence compared with Arabidopsis thaliana. Physiological analyses during seed germination under salt–alkali stress revealed significant changes in malondialdehyde content, proline accumulation, and antioxidant enzyme activities, suggesting activation of oxidative stress defense and osmotic adjustment mechanisms. Transcriptome profiling of seedlings treated with 150 mM salt–alkali solution for 5 and 10 days identified 7,350 differentially expressed genes, including 29 AP2/ERF members responsive to stress. Among them, 13 genes, including *AP2-9, ERF23, ERF15, ERF31, ERF34*, and *DREB21*, were consistently upregulated under both treatments, indicating their potential roles in stress adaptation. qRT-PCR validation further confirmed the sustained upregulation of key genes *AP2-9, ERF23, ERF34, and DREB21*, consistent with transcriptome data. Overall, this study provides the first comprehensive overview of the AP2/ERF gene family in *N. nucifera* and identifies candidate regulators involved in salt–alkali stress responses, offering valuable insights into the molecular mechanisms of stress adaptation and potential genetic resources for breeding salt–alkali tolerant aquatic plants.

## 1 Introduction

The APETALA2/ethylene-responsive factor (AP2/ERF) superfamily represents one of the largest and most evolutionarily conserved transcription factor families in plants, playing pivotal roles in diverse developmental processes and stress adaptation (Jiang et al., 2022; Feng et al., 2020; Licausi et al., 2013; Xing et al., 2021; Yang et al., 2021). As a plant-specific gene family, AP2/ERF members are characterized by the presence of one or two highly conserved AP2 DNA-binding domains, each comprising 60–70 amino acid residues and mediating the regulatory functions by binding to specific cis-elements in target gene promoters (Licausi et al., 2010). Based on structural variations and functional divergence, this superfamily is classified into five major subfamilies: APETALA2 (AP2), dehydration-responsive element-binding (DREB), ethylene-responsive element-binding protein (ERF), related to ABI3/VP1 (RAV), and Soloist (Park et al., 2025; Tan et al., 2025). Among these, the AP2 subfamily members uniquely contain two AP2 domains, whereas DREB and ERF members possess only a single AP2 domain (Bakhtari et al., 2024). The RAV subfamily is distinguished by an additional B3 DNA-binding domain, a feature conserved in other plant-specific transcription factors (Zhang et al., 2024). In contrast, the Soloist subfamily members, despite harboring an AP2 domain, exhibit limited sequence homology with other members, suggesting a distinct evolutionary trajectory (Wang et al., 2024). Given their critical regulatory roles in growth, development, and stress responses, AP2/ERF transcription factors have been extensively studied across plant species (Feng et al., 2025; Bakhtari et al., 2024), offering valuable insights into their molecular mechanisms and potential biotechnological applications.

Under abiotic stresses such as low temperature, drought, and high salinity, AP2/ERF transcription factors play crucial regulatory roles by rapidly activating downstream stress-responsive genes to enhance plant adaptation (Kong et al., 2023; Shu et al., 2015). For instance, in tomato (Solanum lycopersicum), SlERF15 directly binds to and activates the promoters of cold-responsive genes (*CBF1, WRKY6*, and *SAG21-lik*e), thereby improving chilling tolerance, while its knockout significantly compromises cold resistance (Hu et al., 2024). Overexpression of the O*phiopogon japonicus OjERF* gene in tobacco (*Nicotiana tabacum*) upregulates stress-responsive genes, increases chlorophyll and proline accumulation, and enhances the activities of antioxidant enzymes (SOD and CAT), collectively improving drought and salt tolerance (Cong, 2013). In *Medicago falcata*, transcriptomic analyses revealed that the ERF family represents one of the most dynamically regulated transcription factor groups under saline-alkali stress, with 131 *ERF* genes potentially modulating antioxidant systems and ion transport to confer salt tolerance (Chai et al., 2025). Functional studies further demonstrate that *GmERF3* overexpression enhances salt and dehydration tolerance in transgenic plants by activating stress-related genes, whereas in potato (*Solanum tuberosum*) (Zhang et al., 2009), *StERF3* acts as a negative regulator—its silencing improves NaCl tolerance, and overexpression increases salt sensitivity (Tian et al., 2015). Additionally, cotton (*Gossypium hirsutum*) overexpressing *GhERF13.12* exhibits significantly enhanced salt tolerance (Lu et al., 2021), and the wild soybean (*Glycine soja*) *GsERF6*, which shares high sequence homology with rice ERF proteins, improves saline-alkali tolerance in transgenic rice by boosting ROS scavenging, osmotic adjustment, and stress-responsive gene expression (Yu et al., 2016). These findings collectively elucidate the dual characteristics of conservation and functional diversification among AP2/ERF transcription factors in plant stress adaptation, particularly in the context of saline-alkali tolerance.

Lotus (*Nelumbo nucifera* Gaertn.) is an important aquatic plant with high ornamental, nutritional, and ecological value and is widely cultivated worldwide (Yang et al., 2024). However, soil salinization and alkalization have become major environmental constraints limiting its growth, productivity, and geographical distribution (Hassani et al., 2021; Song et al., 2023). In recent years, the adverse effects of salt-alkali stress on both crop and ornamental plant performance have become increasingly prominent due to global climate change and accelerated agricultural land degradation (Zilu et al., 2025; Li et al., 2025b). Although *N. nucifera* generally exhibits moderate salt tolerance, its tolerance capacity varies significantly depending on the cultivar, developmental stage, and environmental conditions (Liu et al., 2014). Under low to moderate salinity (e.g., ≤50 mM NaCl), certain *N. nucifera* cultivars can adapt to stress conditions through physiological mechanisms, including osmotic adjustment, enhanced antioxidant defense, and maintenance of ion homeostasis (Salas-Muñoz et al., 2012). Nevertheless, high salinity levels (>100 mM NaCl) often result in growth inhibition, leaf damage, and reduced flowering (Chen et al., 2013). The adaptive capacity of *N. nucifera* to saline-alkaline environments is closely associated with its underlying molecular regulatory networks (Chen et al., 2013; Jia et al., 2019). Among these, the AP2/ERF transcription factor family plays a pivotal role in regulating plant responses to abiotic stresses (Jia et al., 2019; Kavas et al., 2015). Members of this family can activate or repress the expression of downstream stress-responsive genes, thereby participating in key physiological processes including osmotic regulation, reactive oxygen species (ROS) scavenging, and ion homeostasis (Zhu et al., 2021). Through these mechanisms, AP2/ERF transcription factors contribute to enhanced salt-alkali stress tolerance (Kavas et al., 2015; Bakhtari et al., 2024; Chen Yue et al., 2022). Despite their critical roles in other plant species, a comprehensive characterization of the AP2/ERF gene family in *N. nucifera*, as well as their regulatory mechanisms in salt-alkali stress response, remains largely unexplored.

Although AP2/ERF transcription factors have been extensively studied in model plants and several crops, the systematic characterization and functional implications of this family in *N. nucifera* remain largely unknown, particularly regarding their roles in salt–alkaline stress responses. Therefore, this study aimed to (i) identify and characterize the AP2/ERF gene family in the lotus genome through genome-wide analysis; (ii) investigate their evolutionary relationships, structural characteristics, and potential functional diversification; and (iii) identify salt–alkaline-responsive AP2/ERF genes using physiological assessment and transcriptome profiling during seed germination. The findings of this study provide a comprehensive framework for understanding AP2/ERF-mediated stress adaptation mechanisms in *N. nucifera* and offer potential candidate genes for improving stress resilience in lotus and other aquatic crops.

## 2 Results

### 2.1 Identification of 101 *AP2/ERF* genes in *N. nucifera*

To identify AP2/ERF family members in *N. nucifera*, we performed a BLASTp analysis using *A. thaliana* AtAP2/ERF protein sequences as queries, which led to the identification of 101 AP2/ERF proteins in *N. nucifera.* After removing redundant sequences corresponding to identical genes and alternative transcripts, the *AP2/ERF* genes in *N. nucifera* were systematically named according to their order in the NCBI reference genome assembly (GCA_000365185) and associated annotation (**Supplementary Table 1**). Bioinformatic analysis revealed substantial variation in the physicochemical properties of these proteins. The AP2/ERF proteins ranged from 116 (ERF8) to 679 (AP2-8) amino acids in length (**Table 1**), with corresponding molecular weights (MW) spanning 13.3438 kDa (ERF8) to 74.78307 kDa (AP2-8) (**Table 1**). The predicted isoelectric points (pI) exhibited a broad range from 4.24 (DREB30) to 10.31 (ERF37) (**Table 1**), with 26 AP2/ERF proteins displaying pI values ≥7, reflecting variation in amino acid composition and theoretical net charge under different pH conditions. Protein stability analysis showed that the instability index varied considerably from 33.03 (DREB9) to 85.25 (DREB11) (**Table 1**). Only three proteins (DREB9, ERF37, and AP2-15) exhibited instability indices below 40, which are generally classified as stable under *in vitro* conditions. The aliphatic index ranged from 43.42 (DREB28) to 85.1 (DREB9) (**Table 1**), indicating diversity in predicted aliphatic amino acid composition among family members. Importantly, DREB9 showed a relatively higher aliphatic index compared with other AP2/ERF proteins. All AP2/ERF proteins displayed negative grand average of hydropathicity values (**Table 1**), suggesting an overall hydrophilic tendency based on computational estimation. Thus, these comprehensive analyses demonstrate substantial diversity in the predicted physicochemical characteristics the 101 AP2/ERF proteins in *N. nucifera*.

**Table 1:** Physicochemical properties analysis of AP2/ERF proteins in *N. nucifera*.

LOC ID	Gene ID	Protein length	Molecular weight	Theoretical pI	Instability index	Aliphatic index	Grand average of hydropathicity
LOC104594736	AP2-1	518	55908.6	5.82	52.48	54.86	-0.631
LOC104599345	AP2-2	511	55576.22	5.9	54.72	51	-0.688
LOC104593481	AP2-3	373	41973.48	5.73	56.57	61.8	-0.791
LOC104605418	AP2-4	441	50380.61	5.95	58.01	58.23	-0.873
LOC104589848	AP2-5	347	38896.92	5.96	46.47	61.38	-0.819
LOC104590512	AP2-6	427	47374.15	8.9	51.11	65.64	-0.796
LOC104591238	AP2-7	677	74523.81	6.42	51.78	60.86	-0.653
LOC104609075	AP2-8	679	74783.07	6.32	50.93	61.94	-0.684
LOC104600583	AP2-9	597	66024.11	6.69	50.67	51.96	-0.879
LOC104609334	AP2-10	597	65778.1	6.61	44.59	54.92	-0.827
LOC104611932	AP2-11	540	59130.38	5.99	54.1	65.48	-0.535
LOC104595508	AP2-12	555	61151.88	6.36	55.29	66.5	-0.521
LOC104597438	AP2-13	552	60509.76	6.58	46.84	59.96	-0.646
LOC104613021	AP2-14	542	59725.9	6.25	53.65	61.61	-0.638
LOC104603172	AP2-15	528	57510.29	6.19	39.82	58.05	-0.583
LOC104586961	S1	232	25958.02	9.6	63.86	52.54	-1.019
LOC104601351	RAV1	365	40720.75	9.07	43.42	71.81	-0.624
LOC104603036	RAV2	365	40798.01	8.91	41.9	75.23	-0.565
LOC104591998	RAV3	366	41939.11	6.29	51.01	65.74	-0.716
LOC104598694	RAV4	397	45259.85	8.02	44.81	63.9	-0.661
LOC104595388	ERF1	178	19545.23	9.12	68.23	69.72	-0.533
LOC104592437	ERF2	189	21016.98	8.87	68.52	73.86	-0.519
LOC104603203	ERF3	150	17052.78	5.74	72.93	61.2	-0.759
LOC104603363	ERF4	185	21162.07	8.46	54.74	66.97	-0.687
LOC104589060	ERF5	187	21116.05	8.81	46.91	72.03	-0.59
LOC104610490	ERF6	304	32693.62	6.77	66.18	63.98	-0.51
LOC104598425	ERF7	309	33296.33	6.78	69.99	67.61	-0.488
LOC104610091	ERF8	116	13343.8	5.76	60.63	68.28	-0.871
LOC104610031	ERF9	135	15363.04	7.87	66.15	66.59	-0.813
LOC104600858	ERF10	131	14742.39	5.48	54.64	61.98	-0.792
LOC104600857	ERF11	143	16178.97	5.86	57.16	62.24	-0.928
LOC104610033	ERF12	154	17026.57	5.94	56.75	49.55	-0.945
LOC104610032	ERF13	155	17238.96	6.84	55.32	53.03	-0.866
LOC104610030	ERF14	253	28696.84	5	66.96	57.43	-0.711
LOC104600859	ERF15	230	26117.91	5.22	70.97	58.52	-0.721
LOC104599517	ERF16	281	31224.74	4.93	51.08	68.47	-0.597
LOC104598426	ERF17	357	39885.54	5.29	57.87	67.79	-0.635
LOC104610488	ERF18	337	37547.08	6.04	52.64	66.26	-0.578
LOC104606510	ERF19	235	25911.64	9.33	64.3	56.94	-0.83
LOC104594121	ERF20	235	26042.77	9.63	60.1	52.85	-0.89
LOC104612420	ERF21	175	19498.27	9.77	45.17	62.51	-0.597
LOC104608169	ERF22	157	17037.27	8.88	62.33	67.26	-0.398
LOC104599450	ERF23	159	17308.63	9.26	63.03	68.18	-0.48
LOC104608167	ERF24	242	25672.25	6.87	54.38	56.16	-0.578
LOC104599451	ERF25	237	25339.87	9	52.61	56.54	-0.63
LOC104593077	ERF26	393	43256.04	5.52	71.67	60.84	-0.598
LOC104591971	ERF27	389	43206.75	5.24	67.66	54.7	-0.731
LOC104586349	ERF28	352	38717.89	4.66	68.19	56.62	-0.495
LOC104592717	ERF29	275	29757.34	4.59	59.66	47.96	-0.521
LOC104591899	ERF30	285	30802.72	4.69	60.3	48.67	-0.461
LOC104592746	ERF31	242	27082.86	6.22	57.7	56.07	-0.842
LOC104592013	ERF32	329	36554.9	8.24	62.21	68.57	-0.729
LOC104599945	ERF33	423	45596.9	5.55	62.83	51.96	-0.612
LOC104589605	ERF34	412	44663.78	6.34	69.83	53.81	-0.605
LOC104606467	ERF35	290	32037.35	5.27	62.8	55.97	-0.753
LOC104601789	ERF36	277	30596.62	5.48	48.75	61.37	-0.689
LOC104607321	ERF37	140	15552.68	10.31	36.33	64.79	-0.801
LOC104606213	ERF38	268	29969.72	7.05	53.73	66.64	-0.688
LOC104598889	ERF39	258	28668.25	8.83	41.84	65.47	-0.719
LOC104609267	ERF40	372	41595.75	4.82	49.25	54.06	-0.85
LOC104600466	ERF41	376	42035.61	4.82	44.12	55.56	-0.741
LOC104606214	ERF42	328	36054.07	5.58	55.03	54.54	-0.761
LOC104598888	ERF43	314	34956.39	5.01	50.93	48.82	-0.875
LOC104600304	ERF44	339	37899.97	5.36	52.58	62.65	-0.627
LOC104593412	ERF45	328	37039.61	5.1	57.08	57.68	-0.838
LOC104602974	ERF46	343	38183.9	4.95	72.53	56.36	-0.846
LOC104601805	ERF47	345	38297.43	5.38	62.31	63.04	-0.745
LOC104597658	ERF48	267	29511.56	6.08	40.74	56.97	-0.738
LOC104600784	ERF49	256	28848.1	7.65	52.56	60.59	-0.705
LOC104588536	ERF50	275	29658.4	9.55	43.87	68.84	-0.349
LOC104603385	ERF51	235	25841.13	9.02	65.97	65.57	-0.459
LOC104605948	DREB1	391	42603.53	6.33	59.63	66.21	-0.542
LOC104600117	DREB2	415	45276.34	6.01	65.26	60.7	-0.565
LOC104588890	DREB3	461	51370.89	6.03	68.7	52.97	-0.863
LOC104586457	DREB4	432	47407.59	6.39	58.44	54.7	-0.724
LOC104600346	DREB5	323	35041.3	8.75	49.51	68.64	-0.493
LOC104609420	DREB6	432	47361.72	5.64	57.15	70.05	-0.472
LOC104586003	DREB7	428	46686.08	5.35	55.52	70.47	-0.453
LOC104599845	DREB8	290	31455.48	8.32	54.92	59.59	-0.676
LOC104604994	DREB9	157	17799.26	4.47	33.03	85.1	-0.401
LOC104609654	DREB10	220	24020.65	5.13	75.42	55.14	-0.609
LOC104608486	DREB11	221	24239.93	5.47	85.25	50.86	-0.619
LOC104586477	DREB12	163	18104.27	5.11	47.53	77.85	-0.543
LOC104609294	DREB13	204	21947.44	5.49	47.2	57.45	-0.488
LOC104609295	DREB14	207	22854.08	4.67	42.53	52.42	-0.627
LOC104600498	DREB15	199	22291.38	4.59	52.93	53.97	-0.721
LOC104606875	DREB16	153	16767.69	9.24	50.37	53.66	-0.779
LOC104601725	DREB17	152	16578.44	9.37	55.41	51.51	-0.795
LOC104593944	DREB18	198	21885.39	5.24	59.03	74.04	-0.509
LOC104589184	DREB19	233	25782	6.39	51.51	77.47	-0.404
LOC104604770	DREB20	190	21140.58	6.33	56.91	57.11	-0.753
LOC104604040	DREB21	187	20786.07	5.3	53.38	59.09	-0.711
LOC104612693	DREB22	187	20161.53	4.87	49.11	70.05	-0.405
LOC104596183	DREB23	236	25237.27	4.68	70.03	64.62	-0.505
LOC104586938	DREB24	211	22425.94	5	45.69	70.9	-0.343
LOC104591308	DREB25	182	19673.14	4.7	49.79	76.15	-0.389
LOC104595123	DREB26	242	26559.67	5.18	57.7	66.53	-0.521
LOC104602399	DREB27	282	31059.44	5.27	50.12	67.2	-0.608
LOC104598459	DREB28	275	29959.54	5.27	63.49	43.42	-0.858
LOC104602444	DREB29	225	25028.77	5.19	50.38	63.02	-0.691
LOC104602443	DREB30	147	15965.66	4.24	52.99	70.61	-0.231

Protein length (aa), molecular weight (kDa), theoretical pI, instability index, aliphatic index, and grand average of hydropathicity (GRAVY) were calculated from primary amino acid sequences annotated in the NCBI reference genome (GCA_000365185) using TBtools. The instability index predicts *in vitro* protein stability (values >40 indicate instability), the aliphatic index reflects the relative volume of aliphatic side chains and potential thermostability, and GRAVY values indicate protein hydrophobicity (positive) or hydrophilicity (negative).

### 2.2 Evolutionary analysis of AP2/ERF family genes in *N. nucifera*

**Figure 1:**
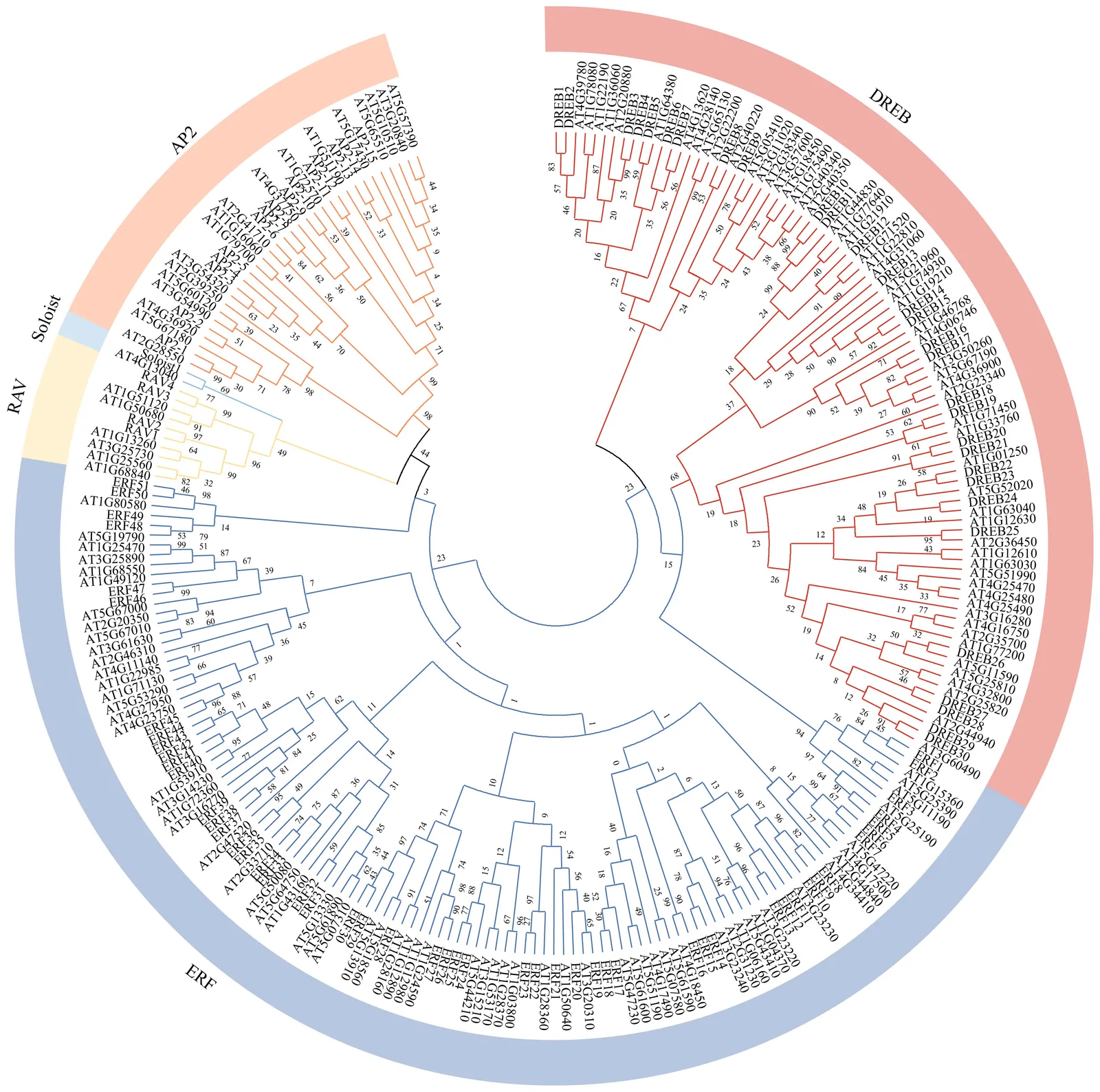
Phylogenetic tree of AP2/ERF gene families in *N. nucifera* and *A.thaliana*. MEGA7 software was employed to construct the phylogenetic tree, and subgroup classification was visualized using different colors.

To investigate the evolutionary relationships within the *AP2/ERF* gene family, we performed a comprehensive phylogenetic analysis using full-length protein sequences from 147 A*. thaliana* and 101 N*. nucifera* AP2/ERF members. The resulting phylogenetic tree clearly delineated these proteins into five well-defined subfamilies (**Figure 1**). The *ERF* subfamily emerged as the most abundant group, comprising 51 members (50.5% of total), followed by the *DREB* subfamily with 30 members (29.7%). The *AP2*, *RAV*, and *Soloist* subfamilies contained 15 (14.85%), 4 (3.96%), and 1 (0.99%) members (**Figure 1**), respectively. Comparative analysis revealed a consistent reduction in gene numbers across all subfamilies in *N. nucifera* compared to *Arabidopsis*, suggesting potential lineage-specific gene loss events during evolution.

Furthermore, synteny analysis identified 117 homologous gene pairs between the two species, with 53 N*. nucifera AP2/ERF* genes showing orthologous relationships to 73 *Arabidopsis* counterparts (**Supplementary Figure 1**; **Supplementary Table S2**). These orthologous pairs were distributed across 35 chromosomal segments, indicating both conserved and divergent evolutionary patterns in the AP2/ERF family between the two species. This systematic characterization not only establishes a framework for understanding AP2/ERF gene family evolution in *N. nucifera* but also highlights key differences in gene family expansion between basal and core eudicots.

### 2.3 Structural characterization and functional diversification of *AP2/ERF* genes in *N. nucifera*

**Figure 2:**
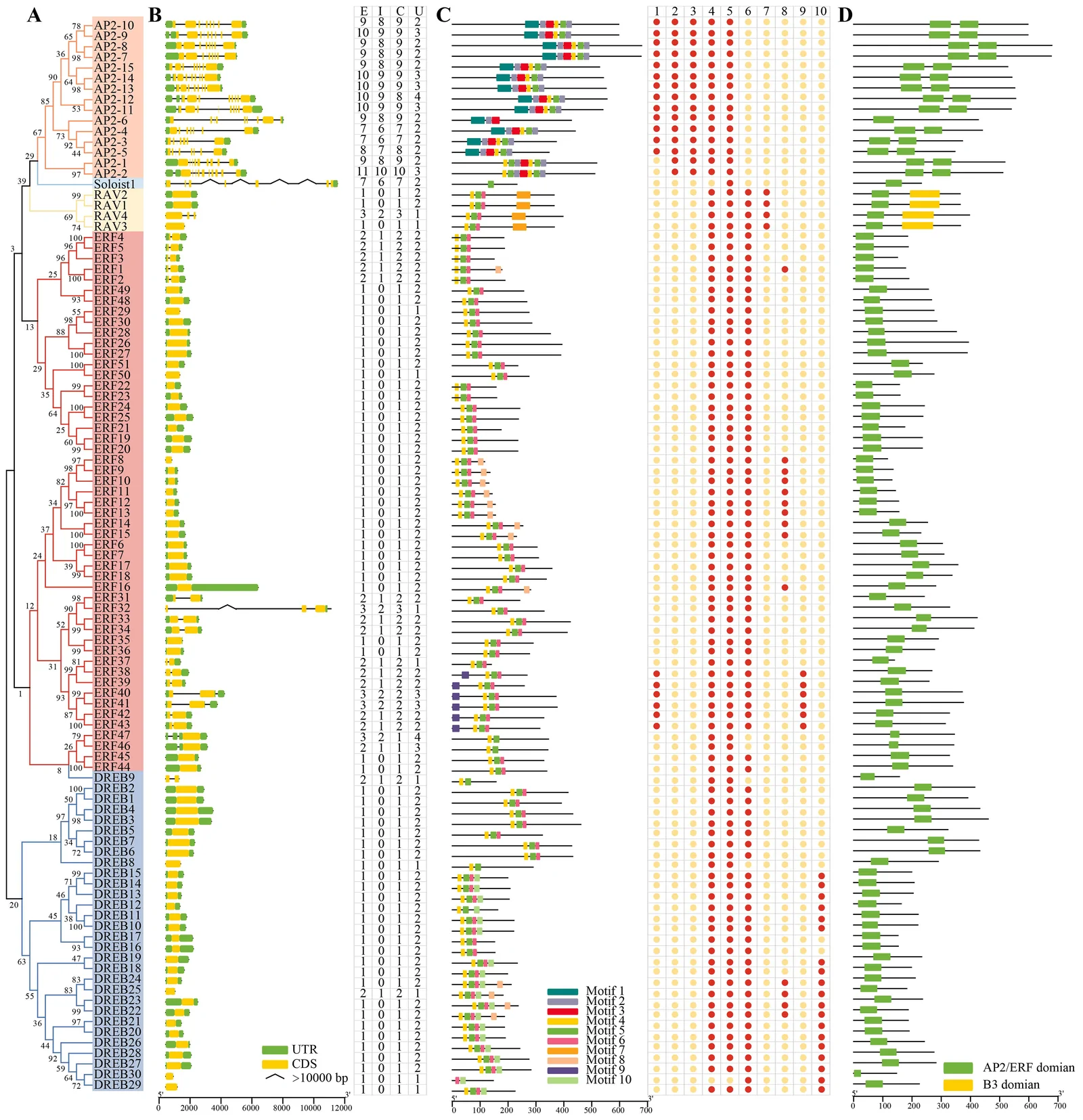
Comparative analysis of phylogenetic relationships, gene structures, conserved motifs, and protein domains of th*e* AP2/ERF gene family in *N. nucifera.* **(A)** Phylogenetic tree of the AP2/ERF gene family, constructed based on amino acid sequences using MEGA7 software. **(B)** Gene structure analysis of 101 *AP2/ERF* gene. Black lines represent introns; green boxes denote 5′ and 3′ untranslated regions (UTRs); yellow boxes represent exons. A table showed the number of exons (E), introns (I), coding sequence (C), and UTRs (U) of *AP2/ERF* genes. **(C)** Conserved motif analysis. Ten conserved motifs were identified within AP2/ERF proteins, with each motif shown in a different color. A table shows whether motifs 1-10 are present in various AP2/ERF protein sequences. Orange dots indicate absence, while red dots indicate presence. **(D)** Conserved domain analysis. Green boxes indicate AP2 domains, and yellow boxes represent B3 domains.

Our comprehensive analysis of gene architecture and conserved domains, revealed critical insights into the functional diversification of AP2/ERF family members in *N. nucifera*. The exon-intron organization exhibited distinct subfamily-specific patterns (**Figure 2A**): The *AP2* subfamily displayed the most complex structures, with five members containing 10 exons and others possessing 7-9 exons. In contrast, the Soloist1 maintained a conserved 7-exon structure. The *RAV* subfamily showed remarkable divergence, with *RAV1-3* being intronless while *RAV4* retained two introns. Within the *ERF* subfamily, 33 genes were single-exon, 14 contained two exons, and *ERF32/40/41/47* uniquely possessed three exons. The *DREB* subfamily predominantly exhibited intron loss, with only *DREB9* and *DREB25* retaining intronic sequences (**Figure 2B**). Analysis of untranslated regions (UTRs) revealed structural variation among *AP2/ERF* genes: 78 genes maintained typical dual-UTR configurations, 8 genes were annotated with three UTRs, and 11 genes contained a single UTR. A few genes, including *ERF47* and *AP2-12*, were annotated with four UTRs; however, these predictions are derived solely from the reference genome annotation and have not been experimentally validated (**Figure 2B**). Protein motif analysis identified 10 conserved motifs (15-50 aa) (**Supplementary Figure 2**). Motifs 4 and 5 were nearly ubiquitous (absent only in DREB30 and Soloist1), while motif 6 was widely distributed across *RAV/ERF/DREB* subfamilies, suggesting their roles as core functional elements (**Figure 2C**). Subfamily-specific motif patterns emerged: the *AP2* subfamily uniquely contained motifs 1-3, with most members possessing duplicate motif 2, while the *RAV* subfamily specifically retained motif 1. The *ERF* subfamily exhibited subgroup specialization, with 10 members containing motif 8 and ERF37-43 possessing motifs 1 and 9. Domain architecture analysis confirmed structural conservation: all AP2 proteins except AP2-6 maintained dual AP2/ERF domains, *RAV* members contained characteristic AP2/ERF-B3 domain combinations, while *ERF* and *DREB* subfamilies possessed single AP2/ERF domains - consistent with established paradigms. Remarkably, *DREB* subfamily members displayed motif simplification, with DREB30 retaining only the minimal motif 6 and motif 10 combination. These findings not only validate conserved structural features but also reveal novel complexities in UTR arrangements, motif combinations, and domain architectures that likely contribute to functional diversification within this crucial transcription factor family. The observed structural variations provide molecular correlates for the evolutionary specialization of AP2/ERF proteins in *N. nucifera*.

### 2.4 Physiological and developmental responses of *N. nucifera* seeds to salt-alkaline stress

**Figure 3:**
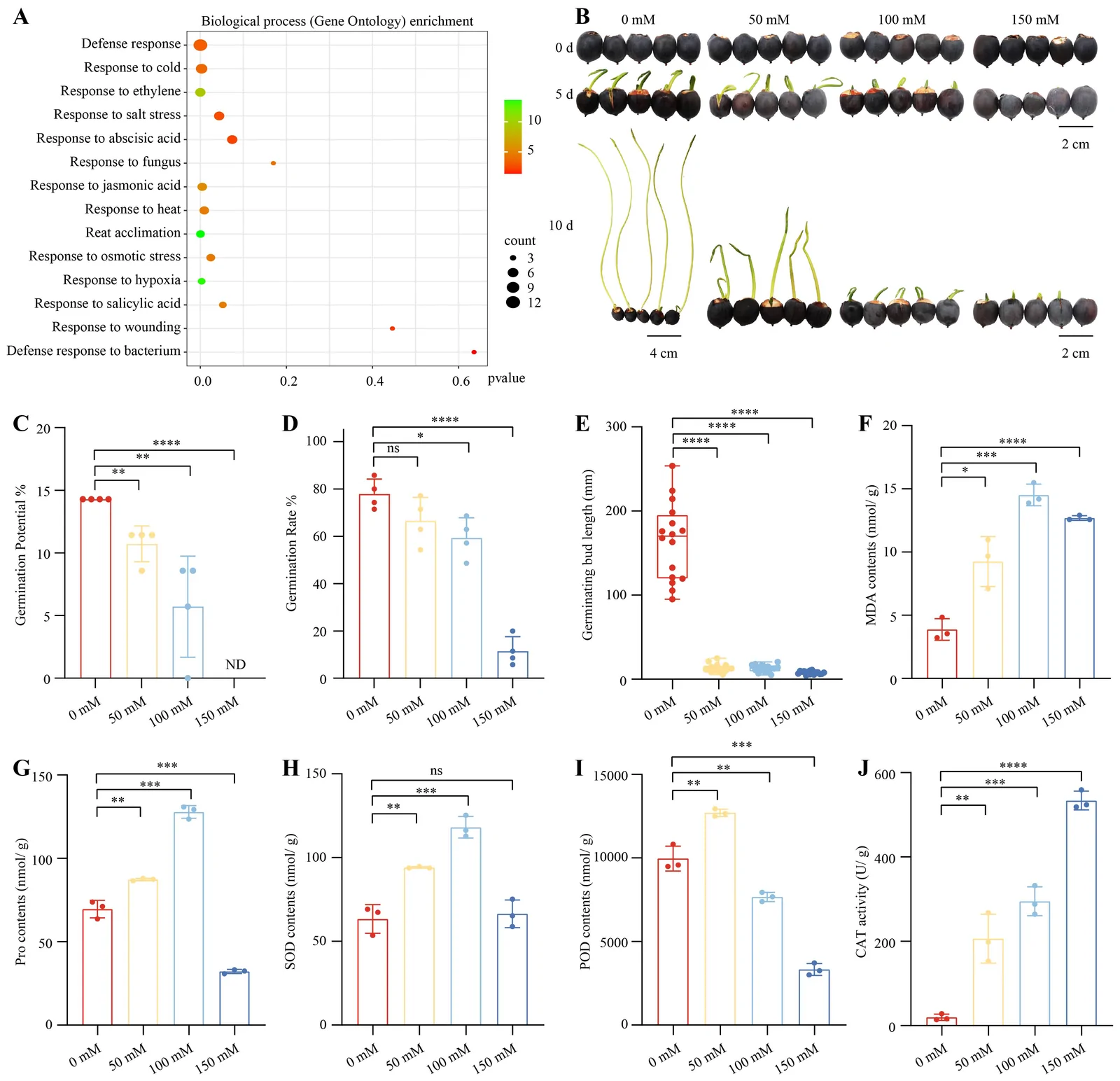
Higher salt-alkaline stress inhibits seedling growth in *N. nucifera*. **(A)** GO enrichment analysis of 147 *Arabidopsis thaliana* AP2/ERF family members. **(B)** Germination phenotypes of *N. nucifera* seeds treated with 0, 50, 100, and 150 mM salt–alkaline solutions at 0, 5, and 10 days. **(C, D)** Germination potential and germination rate of the seeds under various salt-alkaline stress conditions. **(E)** Radicle length of germinated seeds under salt-alkaline stress. **(F–J)** Contents or activities of MDA, proline, SOD, POD, and CAT under different salt–alkaline treatments at 10 days. Statistical significance was determined using one-way ANOVA followed by Tukey’s HSD multiple comparison test. *, **, ***, and **** indicate significance at p ≤ 0.05, 0.01, 0.001, and 0.0001, respectively; ns indicates no significance.

The AP2/ERF transcription factor family plays a pivotal role in plant responses to abiotic stresses, including salinity and alkalinity. Gene Ontology (GO) enrichment analysis of 147 AtAP2/ERF members in *A. thaliana* revealed significant enrichment in both biotic and abiotic stress-responsive pathways, with particularly strong associations with cold and salt stress responses (**Figure 3A**). We hypothesize that AP2/ERF transcription factors in *N. nucifera* may also perform similar biological functions. To elucidate the regulatory role of AP2/ERF transcription factors in salt–alkaline tolerance in *N. nucifera*, we focused on the seed germination stage. This developmental phase is highly sensitive to salt–alkaline stress. Seeds were treated with mixed salt–alkaline solutions (NaCl : NaHCO_3_ = 1:1, molar ratio) at different concentrations (0, 50, 100, and 150 mM). Germination dynamics and physiological responses were then systematically assessed. After 5 days of treatment, seeds in the control (0 mM) and 50 mM groups had successfully germinated and developed visible shoots, whereas shoot elongation was markedly inhibited at 100 mM and almost completely suppressed at 150 mM. By day 10, these differences became more pronounced: shoot lengths reached approximately 100–300 mm in the control group, were moderately reduced at 50 mM, severely inhibited at 100 mM, and nearly arrested at 150 mM (**Figures 3B, E**). Both germination potential and germination rate exhibited a significant negative correlation with increasing salt-alkaline concentration (**Figures 3C, D**). Biochemical analyses revealed that levels of malondialdehyde (MDA), proline (Pro), and superoxide dismutase (SOD) activity increased significantly with salt-alkaline concentrations up to 100 mM, but were markedly reduced at 150 mM (**Figures 3F–H**). Peroxidase (POD) activity peaked at 50 mM, began to decline at 100 mM, and showed no significant difference from the control at 150 mM. In contrast, catalase (CAT) activity consistently declined with increasing salt-alkaline concentration (**Figures 3I, J**). These findings suggest that low to moderate salt–alkaline stress induces protective physiological responses, including enhanced proline accumulation and activation of reactive oxygen species (ROS)-scavenging systems, which may contribute to maintaining cellular homeostasis under adverse conditions.

### 2.5 Identification of key AP2/ERF family members responsive to salt-alkaline stress in *N. nucifera*

**Figure 4:**
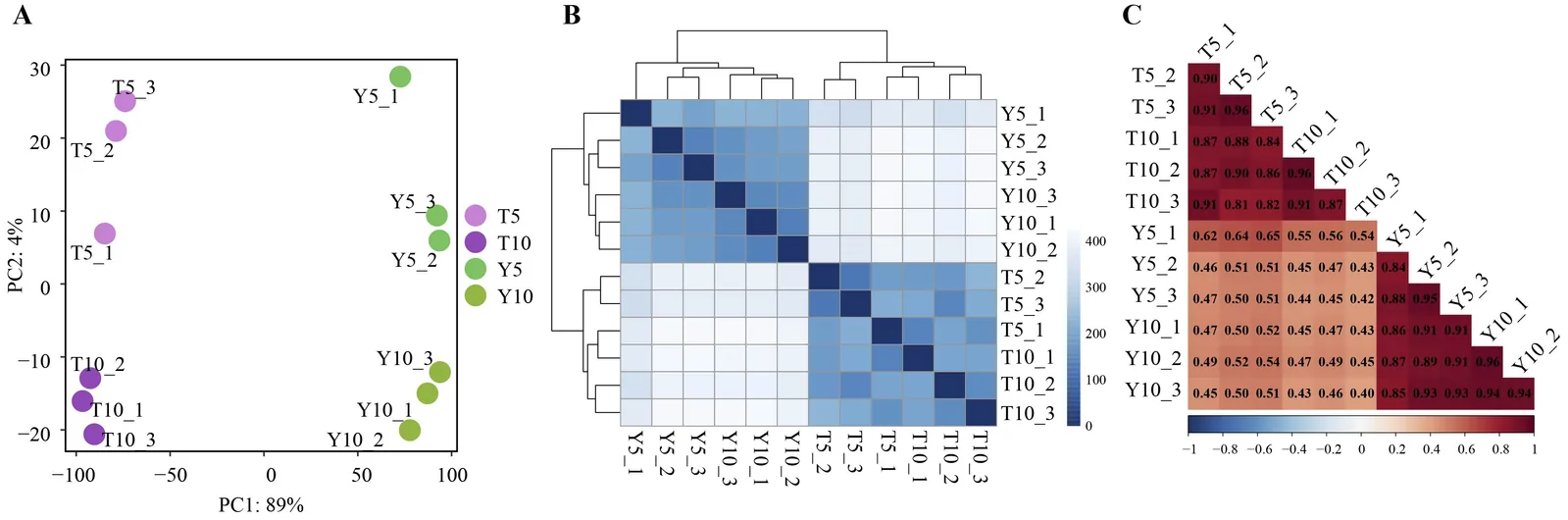
Assessment of sample consistency and reproducibility in RNA-seq data. **(A)** Principal component analysis (PCA) based on normalized gene expression levels (FPKM). **(B)** Hierarchical clustering analysis of RNA-seq samples. **(C)** Pearson correlation analysis among samples. T5, 0 mM treatment for 5 days; Y5, 150 mM treatment for 5 days; T10, 0 mM treatment for 10 days; Y10, 150 mM treatment for 10 days.

The physiological responses demonstrated that 100 mM represented a moderate stress condition, whereas 150 mM induced severe developmental inhibition and physiological changes. Therefore, 150 mM was selected for transcriptome analysis to capture pronounced stress-responsive transcriptional changes and identify key regulatory genes and pathways. To further elucidate the molecular regulatory mechanisms by which the AP2/ERF transcription factor family mediates salt-alkaline stress responses in *N. nucifera* seeds, transcriptome profiling was performed on embryonic tissues collected from seeds after 5 days and on shoot tissues after 10 days of treatment under 0 mM and 150 mM salt–alkaline conditions. Each treatment and time point included three independent biological replicates. The four sample groups were designated as 0 mM-5d, 150 mM-5d, 0 mM-10d, and 150 mM-10d. High-quality RNA samples (RIN ≥ 7.0) were used for library construction and Illumina sequencing. Sequencing data statistics for all 12 samples, including total read, reads mapped, raw and clean read counts, total bases, and Q20/Q30 percentages (**Supplementary Table 3**), demonstrate consistently high sequencing quality across all replicates. Sample consistency and reproducibility were assessed using principal component analysis (PCA) (**Figure 4A**), hierarchical clustering (**Figure 4B**), and Pearson correlation analysis (**Figure 4C**) based on normalized gene expression levels (FPKM). All three analyses showed that biological replicates clustered tightly together, with clear separation between different treatments and time points (**Figure 4**), indicating high reproducibility among replicates and confirming the reliability and robustness of the RNA-seq dataset.

**Figure 5:**
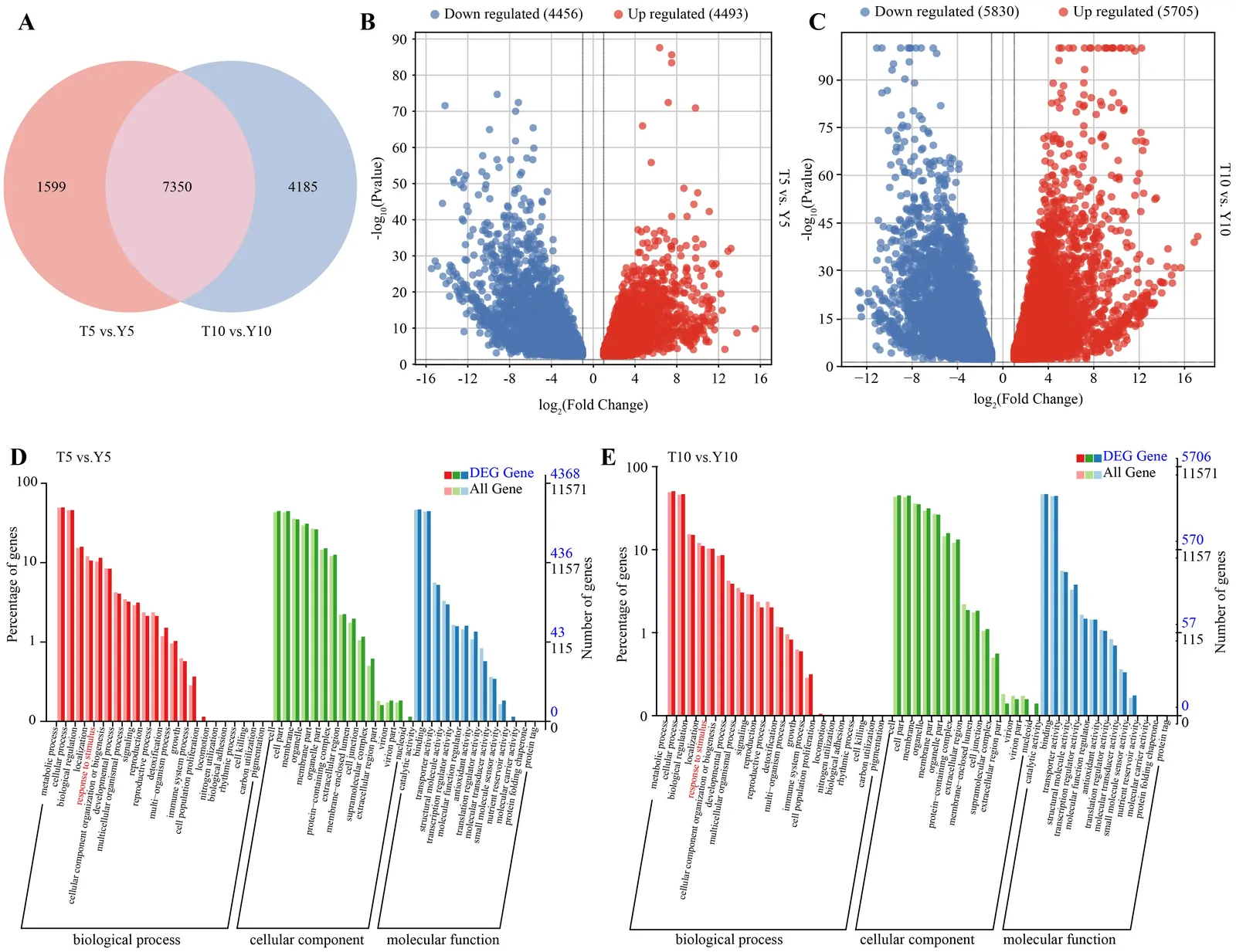
Transcriptomic analysis of *N. nucifera* under salt-alkaline stress. **(A)** Venn diagram showing overlaps of differentially expressed genes (DEGs) among T5, Y5, T10, and Y10. **(B, C)** Volcano plots of DEGs in the T5 vs. Y5 and T10 vs. Y10 Red dots indicate significantly upregulated genes, and blue dots indicate significantly downregulated genes. **(D, E)** GO enrichment analysis of DEGs identified in T5 vs Y5 and T10 vs Y10 comparisons.

Comparative transcriptomic analysis revealed a total of 7,350 differentially expressed genes (DEGs) commonly identified between control and treatment groups (**Figure 4A**; **Supplementary Figure S3**). Specifically, 8,949 DEGs were identified between the 0 mM and 150 mM samples on day 5, including 4,493 upregulated and 4,456 downregulated genes. On day 10, the number of DEGs increased to 11,535, with 5,705 genes upregulated and 5,830 downregulated under salt-alkaline stress (**Figures 5A–C**). Gene Ontology (GO) enrichment analysis showed that these DEGs were significantly enriched in key biological processes such as metabolism, biological regulation, and responses to stimuli (**Figures 5E, F**). KEGG pathway analysis indicated that salt–alkaline stress mainly affected pathways related to energy metabolism, antioxidant defense, osmotic regulation, cell cycle and DNA replication, and protein quality control (**Supplementary Figure 4**), providing a molecular framework for understanding the regulatory roles of AP2/ERF transcription factors under salt–alkaline stress in *N. nucifera*.

**Figure 6:**
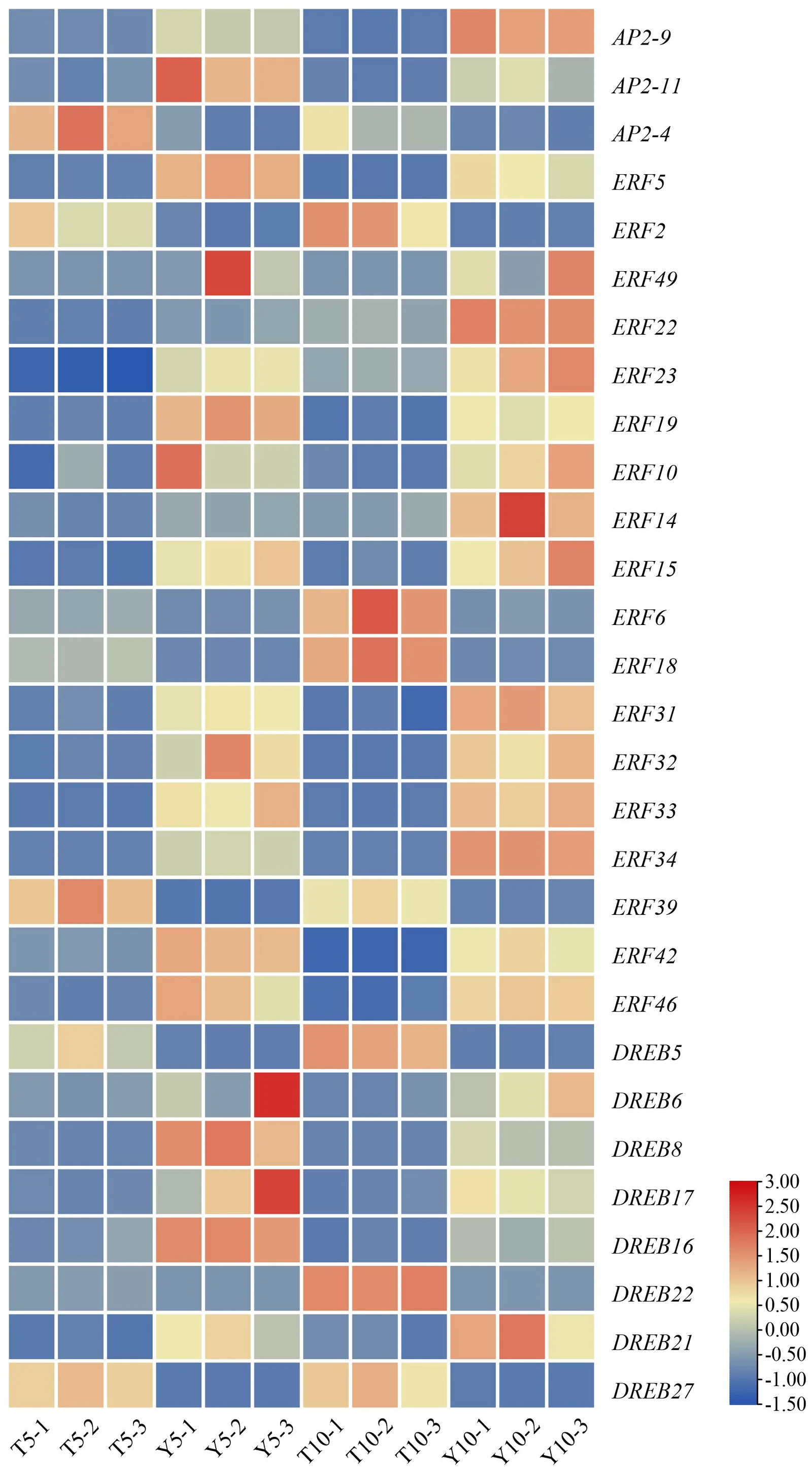
Expression profiles of 29 AP2/ERF transcription factors significantly affected by salt-alkaline stress. The heatmap displays the expression patterns of 29 AP2/ERF transcription factors in response to salt-alkaline treatment in *N. nucifera*, including 3 *AP2* subfamily members, 18 *ERF* subfamily members, and 8 *DREB* subfamily members. The color scale represents normalized expression levels (log_2_-transformed values).

Further screening of the 7,350 common DEGs led to the identification of 29 AP2/ERF transcription factors with significant expression changes (**Figure 5**), comprising 3 *AP2* subfamily members, 18 *ERF* subfamily members, and 8 *DREB* subfamily members (**Figure 6**). Importantly, seven AP2/ERF genes were significantly downregulated under salt–alkaline treatment, suggesting that these genes may be involved in stress-responsive transcriptional regulation. In contrast, 13 *AP2/ERF* genes exhibited sustained upregulation under 150 mM salt-alkaline conditions, indicating their likely involvement in stress adaptation and regulatory processes (**Figure 6**). Among them, *AP2-9*, *ERF23*, *ERF15*, *ERF31*, *ERF34*, and *DREB21* showed particularly strong and consistent induction, suggesting these genes may serve as key regulators in the salt-alkaline stress response network of *N. nucifera* seeds. These findings provide critical insights into the transcriptional regulatory networks governed by the AP2/ERF family and offer valuable clues for dissecting salt-alkaline tolerance mechanisms in *N. nucifera*.

### 2.6 Expression validation of key *AP2/ERF* genes during seed germination of *N. nucifera* under salt–alkaline stress

**Figure 7:**
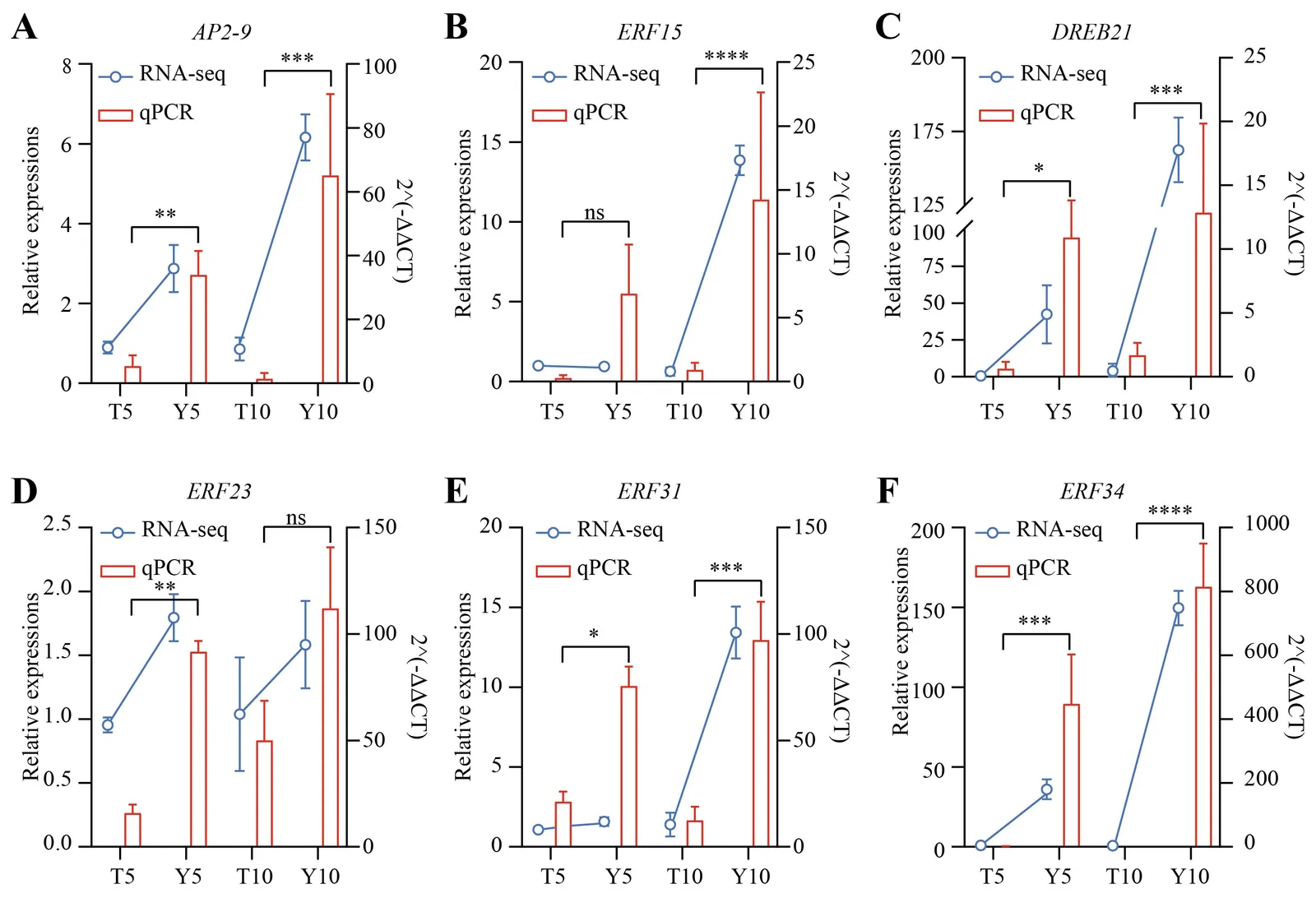
Expression validation of key AP2/ERF genes during seed germination of *Nelumbo nucifera* under salt–alkali stress. Expression patterns of AP2-9 **(A)**, ERF23 **(B)**, ERF15 **(C)**, ERF31 **(D)**, ERF34 **(E)**, and DREB21 **(F)** were analyzed in seeds treated with 0 mM (control) and 150 mM salt–alkaline solutions for 5 and 10 days. The line graphs represent RNA-seq expression levels based on FPKM values, whereas the bar graphs represent qRT-PCR relative expression levels calculated using the 2^-^ΔΔCt method. For qRT-PCR analysis, the corresponding untreated control samples at each time point were used as calibrators and assigned a relative expression value of 1. A dual Y-axis format was used to display RNA-seq and qRT-PCR data with different expression scales. Statistical significance was determined using one-way ANOVA followed by Tukey’s HSD multiple comparison test. *, **, ***, and **** indicate significance at *p* ≤ 0.05, 0.01, 0.001, and 0.0001, respectively; ns indicates no significance.

Based on transcriptome analysis, we identified *AP2-9*, *ERF23*, *ERF15*, *ERF31*, *ERF34*, and *DREB21* as potential key members of the AP2/ERF transcription factor family involved in the response of *N. nucifera* during seed germination under salt–alkali stress. To validate their expression patterns, *N. nucifera* seeds were treated with 0 mM (control) and 150 mM salt–alkaline solution for 5 and 10 days, followed by quantitative real-time PCR (qRT-PCR) analysis. The results showed that under 150 mM salt–alkali stress, the expression levels of *AP2-9*, *ERF23*, *ERF15*, *ERF31*, *ERF34*, and *DREB21* were significantly upregulated compared with the control at both 5 and 10 days of treatment (**Figures 7A–F**). However, when compared with the transcriptome data, only *AP2-9*, *ERF23*, *ERF34*, and *DREB21* exhibited consistent expression trends across both treatments and time points (**Figures 7A, C, D, F**). Significantly, *ERF15* and *ERF31* did not show significant differential expression in the transcriptome data at day 5 of salt–alkaline treatment, yet were markedly upregulated according to the qRT-PCR results (**Figures 7B, E**). This discrepancy suggests that their regulatory mechanisms may be more complex, warranting further investigation into their biological functions and regulatory pathways. Overall, these findings indicate that *AP2-9*, *ERF23*, *ERF34*, and *DREB21* exhibit highly consistent expression patterns between transcriptome and qRT-PCR analyses, supporting their pivotal regulatory roles in the response of *N. nucifera* to salt–alkaline stress during seed germination.

## 3 Discussion

Lotus (*Nelumbo nucifera*) is an important aquatic plant with high ornamental, nutritional, and ecological values, and is widely cultivated worldwide. To elucidate the functional roles of AP2/ERF genes in lotus, the NCBI reference genome assembly (accession No. GCA_000365185) was utilized in this study, and a total of 101 AP2/ERF family members were identified. Previous studies have reported 121 AP2/ERF genes in *N. nucifera* based on the Nelumbo Genome Database (Cao et al., 2021). The difference in gene numbers may result from variations in genome resources, annotation versions, and identification strategies. Since the complete genome sequence and annotation information of the Nelumbo Genome Database are not fully available, direct comparison between the two gene sets remains difficult. Based on phylogenetic analysis, the identified AP2/ERF genes were classified into five subfamilies, with most members belonging to the AP2, ERF, and DREB groups (**Figure 1**). This classification is consistent with previous studies in *A. thaliana* (Xie et al., 2019), *Passiflora edulis* (Luo et al., 2025), cucumber (Hu and Liu, 2011), barley (Bai et al., 2025), and tomato (Chen Y. et al., 2023). The relatively high proportion of ERF and DREB members suggests their potential importance in regulating developmental processes and stress responses. Previous studies have established functional evidence for AP2/ERF genes in lotus, including *NnADAP*-mediated regulation of rhizome morphology and *ERF*-associated responses to abiotic stress (Cao et al., 2021; Xu et al., 2024). Building upon these studies, our work further explored AP2/ERF transcriptional responses during seed germination under salt–alkaline stress through integrated physiological and transcriptomic analyses.

The number of AP2/ERF members varies considerably among plant species, reflecting differences in evolutionary history, genome duplication events, tandem or segmental duplication, and ecological adaptation (Wang et al., 2019; Shigyo et al., 2006). Compared with *A. thaliana* (147 AP2/ERF genes), *N. nucifera* possesses a smaller AP2/ERF family, whereas species such as *Brassica rapa* (291 members), rice (170 members), and maize (214 members) contain expanded AP2/ERF repertoires, which have been associated with whole-genome duplication and lineage-specific gene retention (Song et al., 2013; Rashid et al., 2012; Zhang et al., 2022). Expansion of specific AP2/ERF subgroups, such as DREB/CBF members in *Eucalyptus grandis*, has also been linked to environmental adaptation, highlighting the role of ecological pressures in shaping gene family evolution (Cao et al., 2015). Conversely, the relatively moderate AP2/ERF family size in *N. nucifera* may reflect its distinct evolutionary trajectory and aquatic lifestyle. Studies in Prosopis cineraria demonstrated that expansion of AP2/ERF genes could contribute to drought adaptation through enhanced DNA-binding capacity and stress regulation (Dhiman et al., 2025), suggesting that AP2/ERF family evolution is closely associated with species-specific environmental challenges. Evolutionary analyses further indicate that basal eudicots such as *N. nucifera* retain relatively conserved AP2/ERF configurations compared with core eudicots, reflecting different patterns of diversification (Zumajo-Cardona and Pabón-Mora, 2016; Lan et al., 2023). In addition to gene number variation, AP2/ERF members in *N. nucifera* exhibited considerable diversity in gene structures and protein characteristics. Differences in exon–intron organization and UTR structures may contribute to transcriptional and post-transcriptional regulatory diversification. The variation observed in protein length, isoelectric point (pI), instability index, and hydrophobicity (**Table 1**) indicates functional differentiation at the protein level. Similar physicochemical diversity has been reported among AP2/ERF proteins in wheat, foxtail millet, maize, almond, and Brachypodium (Lata et al., 2014; Chen et al., 2016; Yu et al., 2022; Zhang et al., 2022). Variation in protein charge and stability may influence DNA-binding activity, subcellular localization, and protein–protein interactions (Riaz et al., 2021; Lata et al., 2014). The predominance of unstable AP2/ERF proteins in *N. nucifera* is consistent with the dynamic regulatory characteristics of transcription factors, as observed in crops such as *Brassica napus* and chickpea (Ghorbani et al., 2020; Agarwal et al., 2016). Meanwhile, highly stable members such as *DREB9* may represent candidates involved in sustained transcriptional regulation under prolonged stress conditions (Chen et al., 2016; Kabir et al., 2021). Together, these findings indicate that although *N. nucifera* contains fewer AP2/ERF members than some terrestrial plants, the family exhibits substantial structural and biochemical diversification, which may provide regulatory flexibility during development and salt–alkaline stress responses.

The relationship between structural characteristics of AP2/ERF genes and their functional diversification provides important insights into transcriptional regulation in plants. In *N. nucifera*, distinct structural features were observed among AP2/ERF subfamilies, which may contribute to their functional specialization. The AP2 subfamily exhibited relatively complex gene structures with more introns, potentially providing opportunities for alternative splicing and regulatory diversification, consistent with observations in almond, chickpea, and *Brachypodium distachyon* (Yu et al., 2022; Agarwal et al., 2016; Chen et al., 2016). In contrast, ERF and DREB members frequently displayed fewer introns or intronless structures (**Figure 2B**), a feature also reported in maize, foxtail millet, and wheat, which may facilitate rapid transcriptional activation during environmental stress responses (Lata et al., 2014; Zhang et al., 2022; Rashid et al., 2012). Consistently, the enrichment of ERF and DREB members among salt–alkaline-responsive genes identified in our transcriptome analysis suggests that these structurally simplified genes may contribute to rapid stress-responsive regulation (**Figures 2B**, **6**). Conserved motif analysis further showed that Motifs 4, 5, and 6 were widely distributed among AP2/ERF members, indicating their essential roles in maintaining conserved AP2/ERF domain functions, whereas subgroup-specific motifs, particularly Motifs 1–3 in AP2 members, may contribute to functional divergence through differential protein interactions and downstream regulatory activities (**Figure 2C**). Similar motif-based diversification patterns have been reported in chickpea, soybean, and grapevine, where subgroup-specific motifs are associated with distinct expression patterns and stress responses (Agarwal et al., 2016; Kabir et al., 2021). Notably, this study identified diverse UTR structures in *N. nucifera* AP2/ERF genes, including genes containing three or four UTR regions, such as *ERF47* and *AP2-12* (**Figure 2B**). Although UTR-mediated regulation of AP2/ERF genes remains insufficiently characterized, previous studies in chickpea and tomato have suggested that UTR variation may influence stress-responsive gene expression through regulation of miRNA binding, mRNA stability, and translation efficiency (Agarwal et al., 2016). These UTR patterns were identified based on the NCBI reference genome annotation (GCA_000365185) and therefore require further experimental validation; however, they provide potential insights into the post-transcriptional regulatory complexity of AP2/ERF genes in *N. nucifera*. Together, these structural and regulatory characteristics may contribute to the differential expression and functional diversification of AP2/ERF members during salt–alkaline stress adaptation.

The physiological responses of *N. nucifera* under salt–alkaline stress exhibited a stress intensity-dependent pattern. MDA, proline accumulation, and SOD activity increased under low to moderate stress but declined under 150 mM treatment (**Figures 3F–J**), indicating that severe stress may exceed the effective adaptive capacity and disrupt cellular homeostasis. Transcriptomic analysis further revealed extensive transcriptional reprogramming, with 29 AP2/ERF genes significantly responding to salt–alkaline stress (**Figure 6**). Among them, *DREB21*, *ERF23*, and *ERF34* showed consistent upregulation and were validated by qRT-PCR (**Figure 7**), suggesting their potential involvement in early stress-responsive regulation in *N. nucifera*. Similar AP2/ERF-mediated stress responses have been reported in other species, where members such as *SsERF52* and *SsSoloist4* in sugarcane, *CaERF2* in pepper, *GmERF105* in soybean, and *IbRAP2-12* in sweetpotato were associated with salt tolerance, ROS homeostasis, or osmotic adjustment (Li et al., 2020; Zhao et al., 2025; Li et al., 2023, 2019). In addition, AP2/ERF members in foxtail millet, cotton, and strawberry have been implicated in ABA-related regulation, proline metabolism, and antioxidant responses under abiotic stress (Lata et al., 2014; Lu et al., 2021; Li et al., 2025a; Liu et al., 2021). Together, these findings support the conserved role of AP2/ERF transcription factors in stress adaptation, while the specific regulatory functions of *DREB21*, *ERF23*, and *ERF34* in lotus require further functional validation.

The present study provides the first comprehensive characterization of the AP2/ERF gene family in *N. nucifera*, revealing its structural, evolutionary, and functional diversity. Interestingly, four UTR structures were identified in several AP2/ERF genes (**Figure 2B**), suggesting potential complexity in post-transcriptional regulation, although further experimental validation is required. Similar regulatory variations in untranslated regions have been reported in Arabidopsis and maize, where cis-regulatory changes contribute to transcriptional plasticity and stress responsiveness (Wang et al., 2019). Transcriptomic analysis under salt–alkaline stress identified a set of differentially expressed AP2/ERF genes associated with physiological responses (**Figures 5**, **6**). The physiological transition observed under 100 mM stress, accompanied by enhanced MDA, Pro, and SOD accumulation and increased expression of *DREB21* and *ERF23* (**Figures 3F–H**, **6**, **7**), indicates an active stress-responsive state before severe cellular damage. Similar transcriptional adjustments have been reported in maize and Arabidopsis under abiotic stress conditions (Izadi et al., 2017; Zhang et al., 2022). Functional studies in other species have demonstrated that AP2/ERF members, such as *OsDREB2B* in rice, DREB genes in *Arabidopsis*, and ERF regulators involved in ethylene and ROS pathways, contribute to stress adaptation (Ma et al., 2022; Todaka et al., 2012; Huang et al., 2022; Seok et al, 2022). However, the specific regulatory functions of AP2-9, *DREB21*, *ERF23*, and *ERF34* in *N. nucifera* remain to be experimentally validated. Future studies involving overexpression, gene editing, and analyses of downstream targets using approaches such as ChIP-seq, EMSA, or DAP-seq will be essential to elucidate the regulatory networks underlying AP2/ERF-mediated salt–alkaline stress responses.

Taken together, our findings establish a conceptual framework linking AP2/ERF family characteristics to alkali stress tolerance in *N. nucifera*. Combined with previous studies on lotus development and stress responses, these results suggest that *AP2/ERF* genes may act as integrative regulators by coordinating growth-related processes with environmental adaptation. In particular, the identification of salt–alkaline responsive members such as *DREB21*, *ERF23*, and *ERF34* during germination highlights a stage-dependent regulatory role that complements earlier reports of AP2/ERF involvement in rhizome development and vegetative stress responses. These observations collectively support a model in which the AP2/ERF family contributes to both developmental plasticity and stress resilience across distinct phases of the lotus life cycle.

## 4 Materials and methods

### 4.1 Identification and systematic analysis of *AP2/ERF*

To identify and classify AP2/ERF family members in *N. nucifera*, a combination of conserved domain identification and comparative phylogenetic analysis was performed using *A. thaliana* AP2/ERF proteins (AtAP2/ERFs) as reference sequences. Reference genome sources: The *N. nucifera* genome was obtained from NCBI (GCA_000365185), and the *A. thaliana* genome was obtained from TAIR (TAIR10.58). Candidate AP2/ERF proteins were initially identified based on the presence of the conserved AP2 DNA-binding domain. The AP2 domain was confirmed using the Pfam database (Pfam accession number: PF00847), and only proteins containing the conserved AP2 domain were retained for subsequent analyses. Full-length protein sequences were aligned using ClustalW with default parameters (Thompson et al., 1994). Phylogenetic relationships were reconstructed in MEGA7 using the Neighbor-Joining (NJ) method with 1,000 bootstrap replicates to assess branch support (Kumar et al., 2016). The NJ method was chosen for its computational efficiency and ability to provide a reliable overview of AP2/ERF subfamily relationships; conserved domains and motif analyses were used to corroborate subfamily classification. Conserved motif analysis was performed using the MEME Suite (https://meme-suite.org/meme/) on full-length AP2/ERF protein sequences (Bailey et al., 2015). The analysis parameters were set as follows: maximum number of motifs 10, motif width 15–50 amino acids, any number of repetitions allowed, and an E-value threshold 1e-5. The identified motifs were subsequently visualized using TBtools (Chen C. et al., 2023). The domain organization of the proteins was examined through Conserved Domain Database (CDD) provided by NCBI (https://www.ncbi.nlm.nih.gov/Structure/bwrpsb/bwrpsb.cgi) (Lu et al., 2020). In addition, the gene structure of *N. nucifera* AP2/ERFs was illustrated using TBtools (Chen C. et al., 2023).

### 4.2 Collinearity analysis of *N. nucifera AP2/ERF* genes

Gene duplication events in *N. nucifera* were identified using the Multiple Collinearity Scan toolkit (MCScanX), applying stringent BLASTp criteria with an e-value threshold of less than 1×10^-5^. Collinear blocks were then defined in MCScanX with a maximum gap of 25 genes and a minimum of five gene pairs per block. To investigate the evolutionary dynamics of gene families in *N. nucifera*, a comparative analysis was conducted using *A. thaliana* as a representative model species. Collinear gene pairs between *N. nucifera* and *A. thaliana* were comprehensively detected and visualized using TBtools (Chen C. et al., 2023).

### 4.3 Phenotypic analysis of *N. nucifera* under different saline-alkali concentrations

Uniform, fully developed, plump, and disease-free seeds of *N. nucifera* were selected for the germination experiment. Because lotus seeds possess strong physical dormancy caused by a hard and water-impermeable seed coat, artificial scarification was performed prior to germination to break dormancy. Specifically, the seed coat was mechanically scarified at the concave end opposite the embryo, using pliers or fine sandpaper to create a small opening, while carefully avoiding damage to the embryo. Following scarification, the seeds were soaked in warm water (20–30°C) for dormancy release, with the soaking water replaced once or twice daily until visible imbibition occurred. Next, seeds were treated with mixed salt–alkaline solutions in a 1:1 molar ratio of NaCl : NaHCO_3_, with total concentrations of 0, 50, 100, and 150 mM (i.e., 25, 50, and 75 mM of each component for the 50, 100, and 150 mM treatments, respectively). Each treatment included three replicates, with 35 seeds per replicate. The 35 seeds per replicate were used exclusively for germination-related phenotypic measurements, including germination potential, germination rate, and shoot length. Independent biological samples were prepared separately for transcriptomic analysis, qRT-PCR validation, and physiological measurements. A 1000 mL beaker was used as the germination container, and 500 mL of the respective saline-alkaline solution was added for the stress treatment. The beakers were incubated in a growth chamber under controlled conditions: plants were grown at 25 °C during a 16 h light period and 20 °C during an 8 h dark period. Germination potential, germination rate, and shoot length were measured on days 0, 5, and 10 to evaluate seed germination and early seedling growth under different concentrations.

Germination was defined as visible radicle emergence from the seed coat. Germination potential and germination rate were calculated according to previous studies on *N. nucifera* seed germination (Lim et al., 2012; Kim et al., 2020; Bewley, 1997) using the following equations:

$\text{Germination potential }(\%)=\frac{\text{Total number of germinated seeds at day }5}{\text{Total number of tested seeds}}\times 100$ $\text{Germination rate }(\%)=\frac{\text{Total number of germinated seeds at day }10}{\text{Total number of tested seeds}}\times 100$Three independent biological replicates were performed for each treatment, and the mean values were used for subsequent statistical analyses.

### 4.4 Determination of MDA, proline, and antioxidant enzyme activities (SOD, POD, and CAT)

To evaluate the physiological responses of *N. nucifera* under saline-alkali stress, the contents of malondialdehyde (MDA), proline (Pro), and the activities of antioxidant enzymes, including superoxide dismutase (SOD), peroxidase (POD), and catalase (CAT), were measured using commercial assay kits purchased from Solarbio (Beijing, China), following the manufacturer’s instructions. The assay kits used in this study included the MDA assay kit (BC0020), proline assay kit (BC0295), SOD assay kit (BC0170), POD assay kit (BC0095), and CAT assay kit (BC0205). For physiological measurements, independent biological samples were collected from the same treatment conditions. Approximately 0.1 g of fresh shoot tissue was collected from each sample for biochemical analysis. When insufficient tissue was available under severe stress conditions, the sample amount was recorded and normalized according to fresh weight or total protein content. For each sample, fresh tissues were homogenized using the corresponding extraction solution provided in the commercial assay kits, following the manufacturer’s protocols. The same extraction procedure was applied to all samples to minimize technical variation. The extraction procedure was performed using the same protocol for all samples. To correct for variation in sample quantity, MDA and proline contents were calculated and expressed on a fresh weight basis, whereas antioxidant enzyme activities were normalized to total protein content. The supernatants were collected for subsequent biochemical analyses. MDA content: Measured by the thiobarbituric acid (TBA) reaction (Lian et al., 2023). Absorbance was read at 532 nm and corrected at 600 nm. Results are expressed as nmol per gram fresh weight (nmol/g FW). Proline content: Determined using the acid ninhydrin method. Absorbance was recorded at 520 nm, and Pro concentration was calculated from a standard curve. Values are expressed as nmol/g FW. SOD activity: Assayed based on the inhibition of nitroblue tetrazolium (NBT) photoreduction (Wang et al., 2022). Absorbance was measured at 560 nm. One unit of SOD activity is defined as the amount of enzyme causing 50% inhibition of NBT reduction (Liu et al., 2023). Activity is expressed as nmol/g protein. POD activity: Determined using guaiacol as a substrate. Absorbance at 470 nm was monitored, and POD activity was defined as the change in absorbance of 0.01 per minute (Hu et al., 2023). Values are expressed as nmol/g protein. CAT activity: Measured by monitoring H_2_O_2_ decomposition at 405 nm (Zhang et al., 2018). One unit of CAT activity corresponds to the decomposition of 1 μmol H_2_O_2_ per minute. Results are expressed as U/g protein.All enzyme activities were normalized to total protein content, which was measured using the Bradford assay. Each treatment included three biological replicates, with each measurement performed in triplicate to ensure reproducibility.

### 4.5 Transcriptome sequencing and differential expression analysis

To investigate the transcriptomic responses of *N. nucifera* shoots under salt-alkaline stress, plants were subjected to 0 mM (control) and 150 mM salt-alkaline treatments, and shoot tissues were harvested at 5 and 10 days post-treatment. Transcriptome samples were obtained from independent biological replicates rather than from the 35 seeds used for phenotypic assessment. At each sampling point, three independent biological replicates were collected, with each replicate consisting of pooled embryonic plumule or shoot tissues from multiple seedlings to obtain sufficient RNA quantity and quality for library construction. After 5 days of treatment, embryonic plumules were carefully dissected from seeds under each treatment condition and collected for RNA extraction, whereas shoot tissues were harvested after 10 days of treatment. Each treatment at each time point consisted of three independent biological replicates. A total of four treatment groups were established: 0 mM-5d (T5), 150 mM-5d (Y5), 0 mM-10d (T10), and 100 mM-10d (Y10), with each sample labeled accordingly as 0 mM-5d-T5, 100 mM-5d-Y5, 0 mM-10d-T10, and 150 mM-10d-Y10. Total RNA was extracted from the collected embryonic plumule or shoot tissues using a Plant RNA Isolation Kit (e.g., TIANGEN, China), following the manufacturer’s instructions. RNA quality and concentration were assessed using a NanoDrop spectrophotometer (Thermo Fisher Scientific, USA), and integrity was evaluated by Agilent 2100 Bioanalyzer (Agilent Technologies, USA). High-quality RNA samples (RIN ≥ 7.0) were used for library construction and sequencing. RNA-seq library preparation and sequencing were performed by Beijing Genomics Biotechnology Co., Ltd (Beijing, China). Libraries were constructed using the NEBNext^®^ Ultra™ RNA Library Prep Kit for Illumina^®^ (New England Biolabs, USA), and sequencing was carried out on the Illumina NovaSeq 6000 platform to generate 150 bp paired-end reads.Clean reads were then aligned to the *N. nucifera* reference genome (NCBI accession: GCA_000365185) using HISAT2 (Kim et al., 2019). This reference genome assembly is accompanied by a comprehensive gene annotation set containing predicted gene models, coding sequences, exon–intron boundaries, and untranslated regions, providing a reliable foundation for transcriptome alignment and downstream expression analysis. Gene-level raw read counts were obtained using featureCounts (Liao et al., 2014). Differential expression analysis was performed using DESeq2 in R based on raw read counts (Love et al., 2014). The log_2_ fold change (log_2_FC) was calculated as the logarithm base 2 of the normalized expression ratio between the treatment and control groups. Genes with |log_2_FC| ≥ 2 and an adjusted *p*-value < 0.05 were considered differentially expressed. Gene expression levels were additionally normalized as fragments per kilobase of transcript per million mapped reads (FPKM) for expression visualization purposes, such as heatmaps and expression profile comparisons. Gene Ontology (GO) and Kyoto Encyclopedia of Genes and Genomes (KEGG) enrichment analyses of DEGs were conducted using the clusterProfilerpackage in R (Yu et al., 2012).

### 4.6 Quantitative RT-PCR analysis

The *AP2-9*, *ERF23*, *ERF15*, *ERF31*, *ERF34*, and *DREB21* genes were selected for quantitative real-time PCR (qRT-PCR) analysis. Gene-specific primers were designed using Primer 5, and primer specificity was confirmed by melting curve analysis. *Actin* was used as the internal reference gene (**Supplementary Table 1**; Song et al., 2024). qRT-PCR was conducted with a Bio-Rad CFX Manager system using SYBR Green Master Mix. Each 10 µL reaction mixture contained 5 µL of SYBR Green Master Mix, 0.4 µL of each primer, 2 µL of cDNA template, and 2.2 µL of nuclease-free water. The amount of cDNA template was selected based on preliminary optimization to ensure reliable amplification and minimize potential effects of excessive template input. The amplification program consisted of an initial denaturation at 95°C for 5 min, followed by 40 cycles of 95°C for 10 s and 60°C for 30 s. A melting curve was generated from 60°C to 90°C to verify amplification specificity. Only primer pairs producing a single specific melting peak were used for subsequent expression analysis. Each sample was analyzed with three independent biological replicates, each measured with three technical replicates. Relative gene expression levels were calculated using the 2^-^ΔΔCt method (Livak and Schmittgen, 2001). The ΔCt value was calculated as Ct (target gene) – Ct (reference gene), and the ΔΔCt value was calculated as ΔCt (treatment sample) – ΔCt (control sample). The relative expression level was determined as 2^-^ΔΔCt, with the corresponding untreated control sample at each sampling time point used as the calibrator and assigned a relative expression value of 1. Therefore, all salt–alkali-treated samples were expressed as fold changes relative to their corresponding controls. The calculated expression values were used for subsequent statistical analysis.

### 4.7 Statistical analysis

Statistical analyses were performed using GraphPad Prism (version 10). Data are presented as mean ± standard deviation (SD) from three independent biological replicates. Differences among multiple treatment groups were evaluated using one-way analysis of variance (ANOVA). When significant differences were detected, Tukey’s honestly significant difference (HSD) test was performed for multiple comparisons. A p-value ≤ 0.05 was considered statistically significant.

## 5 Conclusion

This study presents a comprehensive analysis of the AP2/ERF transcription factor family in *N. nucifera*, integrating genome-wide identification, structural and evolutionary characterization, and transcriptomic profiling under salt-alkaline stress conditions. A total of 101 *AP2/ERF* genes were identified and classified into five subfamilies, showing considerable diversity in gene structure, motif composition, and physicochemical properties. Phylogenetic and syntenic comparisons with *A. thaliana* revealed both conserved and species-specific evolutionary patterns. Physiological and biochemical assays indicated that salt-alkaline stress markedly affects seed germination and growth, accompanied by dynamic changes in oxidative stress-related indices. Transcriptome analyses revealed 29 *AP2/ERF* genes with differential expression under stress, among which 13 genes, including *AP2-9*, *ERF23*, *ERF15*, *ERF31*, *ERF34*, and *DREB21*, exhibited sustained upregulation, suggesting their potential involvement in the salt-alkaline stress response. Subsequent qRT-PCR validation confirmed the expression trends of *AP2-9*, *ERF23*, *ERF34*, and *DREB21* in both 5-day and 10-day treatments, supporting their putative roles in transcriptional regulation under stress. Overall, these results provide valuable insights into the molecular responses of *N. nucifera* to salt-alkaline conditions and establish a foundation for future functional studies aimed at improving stress resilience in lotus and other aquatic crops.

## Data Availability

The datasets generated and analysed during the current study are available in the NCBI SRA repository (BioProject ID: PRJNA1452485).

## References

[B1] Agarwal G. Garg V. Kudapa H. Doddamani D. Pazhamala L. T. Khan A. W. . (2016). Genome-wide dissection of AP2/ERF and HSP90 gene families in five legumes and expression profiles in chickpea and pigeonpea. Plant Biotechnol. J. 14, 1563–1577. doi: 10.1111/pbi.12520 26800652 PMC5066796

[B2] Bai Y. Luo X. Qian W. Geng X. Bi X. Zhang Y. (2025). Identification and analysis of the AP2/ERF gene family in barley based on pan-genome and pan-transcriptome. J. Agric. Food Chem. 73, 18448–18455. doi: 10.1021/acs.jafc.5c05138 40657825

[B3] Bailey T. L. Johnson J. Grant C. E. Noble W. S. (2015). The MEME suite. Nucleic Acids Res. 43, W39–W49. doi: 10.1093/nar/gkv416 25953851 PMC4489269

[B4] Bakhtari B. Razi H. Alemzadeh A. Dadkhodaie A. Moghadam A. (2024). Identification and characterization of the quinoa AP2/ERF gene family and their expression patterns in response to salt stress. Sci. Rep. 14, 29529. doi: 10.1038/s41598-024-81046-1 39604476 PMC11603269

[B5] Bewley J. D. (1997). Seed germination and dormancy. Plant Cell 9, 1055–1066. doi: 10.1105/tpc.9.7.1055 12237375 PMC156979

[B6] Cao D. Lin Z. Huang L. Damaris R. N. Yang P. (2021). Genome-wide analysis of AP2/ERF superfamily in lotus (Nelumbo nucifera) and the association between NnADAP and rhizome morphology. BMC Genomics 22, 171. doi: 10.1186/s12864-021-07473-w 33750315 PMC7945336

[B7] Cao P. B. Azar S. SanClemente H. Mounet F. Dunand C. Marque G. . (2015). Genome-wide analysis of the AP2/ERF family in Eucalyptus grandis: an intriguing over-representation of stress-responsive DREB1/CBF genes. PLoS One 10, e0121041. doi: 10.1371/journal.pone.0121041 25849589 PMC4388522

[B8] Chai H. Wang X. Yang Z. Li S. Xu Y. Wu Y. . (2025). Comparative transcriptome analysis of differentially expressed genes of Medicago falcata L. breeding lines response to saline-alkaline stress. BMC Plant Biol. 25, 623. doi: 10.1186/s12870-025-06599-3 40360985 PMC12070579

[B9] Chen C. Wu Y. Li J. Wang X. Zeng Z. Xu J. . (2023). TBtools-II: a "one for all, all for one" bioinformatics platform for biological big-data mining. Mol. Plant 16, 1733–1742. doi: 10.1016/j.molp.2023.09.010 37740491

[B10] Chen L. Han J. Deng X. Tan S. Li L. Li L. . (2016). Expansion and stress responses of AP2/EREBP superfamily in Brachypodium distachyon. Sci. Rep. 6, 21623. doi: 10.1038/srep21623 26869021 PMC4751504

[B11] Chen L. Wang Q. Q. Zhou L. Ren F. Li D. D. Li X. B. (2013). Arabidopsis CBL-interacting protein kinase (CIPK6) is involved in plant response to salt/osmotic stress and ABA. Mol. Biol. Rep. 40, 4759–4767. doi: 10.1007/s11033-013-2572-9 23649767

[B12] Chen Y. Yang H. Tang B. Li F. Xie Q. Chen G. Hu Z. (2023). The AP2/ERF transcription factor SlERF.J2 functions in hypocotyl elongation and plant height in tomato. Plant Cell Rep. 42, 371–383. doi: 10.1007/s00299-022-02963-x 36512035

[B13] Chen Yue S. M.-Z. Jia B.-W. Leng Y. Sun X.-L. (2022). Research progress regarding the function and mechanism of rice AP2/ERF transcription factor in stress response. Acta Agron. Sin. 48, 781–790. doi: 10.3724/sp.j.1006.2022.12026

[B14] Cong L. (2013). Identification and characterization of transcription factor QjERF gene from mondo grass. (doctoral dissertation). (Beijing: Beijing Forestry University).

[B15] Dhiman V. Marik D. Amrita Shekhawat R. S. Swain A. K. Dey A. . (2025). AP2/ERF transcription factor orthologs of the desert tree Prosopis cineraria show higher copy number and DNA-binding affinity than drought-sensitive species. J. Plant Growth Regul. 44, 2139–2163. doi: 10.1007/s00344-024-11532-3 30311153

[B16] Feng D. He S. Chung J. P. (2025). Isolation of the AP2/ERF transcription factor CaERF14 in pepper and functional characterization under salinity and dehydration stress. Sci. Rep. 15, 19726. doi: 10.1038/s41598-025-03808-9 40473726 PMC12141531

[B17] Feng K. Hou X. L. Xing G. M. Liu J. X. Duan A. Q. Xu Z. S. . (2020). Advances in AP2/ERF super-family transcription factors in plant. Crit. Rev. Biotechnol. 40, 750–776. doi: 10.1080/07388551.2020.1768509 32522044

[B18] Ghorbani R. Zakipour Z. Alemzadeh A. Razi H. (2020). Genome-wide analysis of AP2/ERF transcription factors family in Brassica napus. Physiol. Mol. Biol. Plants 26, 1463–1476. doi: 10.1007/s12298-020-00832-z 32647461 PMC7326749

[B19] Hassani A. Azapagic A. Shokri N. (2021). Global predictions of primary soil salinization under changing climate in the 21st century. Nat. Commun. 12, 6663. doi: 10.1038/s41467-021-26907-3 34795219 PMC8602669

[B20] Hu L. Liu S. (2011). Genome-wide identification and phylogenetic analysis of the ERF gene family in cucumbers. Genet. Mol. Biol. 34, 624–633. doi: 10.1590/s1415-47572011005000054 22215967 PMC3229118

[B21] Hu X. H. Shen S. Wu J. L. Liu J. Wang H. He J. X. . (2023). A natural allele of proteasome maturation factor improves rice resistance to multiple pathogens. Nat. Plants 9, 228–237. doi: 10.1038/s41477-022-01327-3 36646829

[B22] Hu C. Wang M. Zhu C. Wu S. Li J. Yu J. Hu Z. (2024). A transcriptional regulation of ERF15 contributes to ABA-mediated cold tolerance in tomato. Plant Cell Environ. 47, 1334–1347. doi: 10.1111/pce.14816 38221812

[B23] Huang Y. Liu L. Hu H. Tang N. Shi L. Xu F. . (2022). Arabidopsis ERF012 is a versatile regulator of plant growth, development and abiotic stress responses. Int. J. Mol. Sci. 23, 6841. doi: 10.1155/2013/432836 35743283 PMC9224505

[B24] Im M. H. Kim B. W. Park Y. S. Yang S. Y. Song C. E. Heo B. G. (2012). Effects of scarification, temperature and sulfuric acid treatments on seed germination of white lotus (Nelumbo nucifera). Korean J. Plant Resour. 25, 7–13.

[B25] Izadi F. Nikfekr R. Soorni J. (2017). Transcription factors-microRNAs regulatory network in response to multiple stresses in Arabidopsis thaliana. Plant Omics 10, 183–189. doi: 10.21475/poj.10.04.17.pne516

[B26] Jia X. M. Zhu Y. F. Hu Y. Zhang R. Cheng L. Zhu Z. L. . (2019). Integrated physiologic, proteomic, and metabolomic analyses of Malus halliana adaptation to saline–alkali stress. Hortic. Res. 6, 91. doi: 10.1038/s41438-019-0172-0 31645949 PMC6804568

[B27] Jiang Q. Wang Z. Hu G. Yao X. (2022). Genome-wide identification and characterization of AP2/ERF gene superfamily during flower development in Actinidia eriantha. BMC Genomics 23, 650. doi: 10.1186/s12864-022-08871-4 36100898 PMC9469511

[B28] Kabir S. M. T. Hossain M. S. Bashar K. K. Honi U. Ahmed B. Emdad E. M. . (2021). Genome-wide identification and expression profiling of AP2/ERF superfamily genes under stress conditions in dark jute (Corchorus olitorius L.). Ind. Crop Prod. 166, 113469. doi: 10.1016/j.indcrop.2021.113469 38826717

[B29] Kavas M. Kizildogan A. Gökdemir G. Baloglu M. C. (2015). Genome-wide investigation and expression analysis of AP2-ERF gene family in salt tolerant common bean. Excli J. 14, 1187–1206. doi: 10.17179/excli2015-600 27152109 PMC4849109

[B30] Kim D. Paggi J. M. Park C. Bennett C. Salzberg S. L. (2019). Graph-based genome alignment and genotyping with HISAT2 and HISAT-genotype. Nat. Biotechnol. 37, 907–915. doi: 10.1038/s41587-019-0201-4 31375807 PMC7605509

[B31] Kong L. Song Q. Wei H. . (2023). The AP2/ERF transcription factor PtoERF15 confers drought tolerance via JA-mediated signaling in Populus. New Phytol. 240, 1848–1867. 37691138 10.1111/nph.19251

[B32] Kumar S. Stecher G. Tamura K. (2016). MEGA7: molecular evolutionary genetics analysis version 7.0 for bigger datasets. Mol. Biol. Evol. 33, 1870–1874. doi: 10.1093/molbev/msw054 27004904 PMC8210823

[B33] Lan M. J. Hou M. Xiao M. Q. Li C. Pan H. Zhang Y. G. . (2023). Research progress of AP2/ERF transcription factors participating in plant secondary metabolism and stress response. J. Plant Genet. Resour. 24, 1223–1235. doi: 10.13430/j.cnki.jpgr.20230322001

[B34] Lata C. Mishra A. K. Muthamilarasan M. Bonthala V. S. Khan Y. Prasad M. (2014). Genome-wide investigation and expression profiling of AP2/ERF transcription factor superfamily in foxtail millet (Setaria italica L.). PloS One 9, e113092. doi: 10.1371/journal.pone.0113092 25409524 PMC4237383

[B35] Li P. Chai Z. Lin P. Huang C. Huang G. Xu L. . (2020). Genome-wide identification and expression analysis of AP2/ERF transcription factors in sugarcane (Saccharum spontaneum L.). BMC Genomics 21, 685. doi: 10.1186/s12864-020-07076-x 33008299 PMC7531145

[B36] Li X. Wang L. Wang H. Hao R. Gao L. Cui H. . (2025b). Dynamic physiology and transcriptomics revealed the alleviation effect of melatonin on Reaumuria trigyna under continuous alkaline salt stress. Front. Plant Sci. 15, 2024. doi: 10.3389/fpls.2024.1486436 39906237 PMC11790669

[B37] Li W. Zhang W. Li H. Yao A. Ma Z. Kang R. . (2025a). Overexpression of a Fragaria × ananassa AP2/ERF transcription factor gene (FaTINY2) increases cold and salt tolerance in Arabidopsis thaliana. Int. J. Mol. Sci. 26, 2109. doi: 10.3390/ijms26052109 40076731 PMC11900429

[B38] Li Y. Zhang H. Zhang Q. Liu Q. Zhai H. Zhao N. . (2019). An AP2/ERF gene, IbRAP2-12, from sweetpotato is involved in salt and drought tolerance in transgenic Arabidopsis. Plant Sci. 281, 19–30. doi: 10.1016/j.plantsci.2019.01.009 30824052

[B39] Li L. Zhu Z. Liu J. Zhang Y. Lu Y. Zhao J. . (2023). Transcription factor GmERF105 negatively regulates salt stress tolerance in Arabidopsis thaliana. Plants (Basel) 12, 3007. doi: 10.21203/rs.3.rs-2640891/v1 37631217 PMC10459988

[B40] Lian Q. Liu W. Ma D. Liang Z. Tang Z. Cao J. . (2023). Precisely orientating atomic array in one-dimension tellurium microneedles enhances intrinsic piezoelectricity for an efficient piezo-catalytic sterilization. ACS Nano 17, 8755–8766. doi: 10.1021/acsnano.3c02044 37070712

[B41] Liao Y. Smyth G. K. Shi W. (2014). featureCounts: an efficient general purpose program for assigning sequence reads to genomic features. Bioinformatics 30, 923–930. doi: 10.1093/bioinformatics/btt656 24227677

[B42] Licausi F. Giorgi F. M. Zenoni S. Osti F. Pezzotti M. Perata P. (2010). Genomic and transcriptomic analysis of the AP2/ERF superfamily in Vitis vinifera. BMC Genomics 11, 719. doi: 10.1186/1471-2164-11-719 21171999 PMC3022922

[B43] Licausi F. Ohme-Takagi M. Perata P. (2013). APETALA2/Ethylene Responsive Factor (AP2/ERF) transcription factors: mediators of stress responses and developmental programs. New Phytol. 199, 639–649. doi: 10.1111/nph.12291 24010138

[B44] Lim S. H. Kim S. H. Park J. J. Park Y. S. Dhungana S. K. Kim I. D. . (2020). Quality characteristics and antioxidant activities of lotus (*Nelumbo nucifera* Gaertn.) sprouts grown under different conditions. Korean J. Plant Res. 33, 666–674. doi: 10.7732/kjpr.2020.33.6.666

[B45] Liu R. Shi H. Wang Y. Chen S. Deng J. Liu Y. . (2014). Comparative physiological analysis of lotus (Nelumbo nucifera) cultivars in response to salt stress and cloning of NnCIPK genes. Sci. Hortic. 173, 29–36. doi: 10.1016/j.scienta.2014.04.032 38826717

[B46] Liu J. X. Wu B. Feng K. Li M. Y. Duan A. Q. Shen D. . (2021). A celery transcriptional repressor AgERF8 negatively modulates abscisic acid and salt tolerance. Mol. Genet. Genomics 296, 179–192. doi: 10.1007/s00438-020-01738-x 33130909

[B47] Liu C. Zou Q. Tang H. Liu J. Zhang S. Fan C. . (2023). Melanin nanoparticles alleviate sepsis-induced myocardial injury by suppressing ferroptosis and inflammation. Bioact. Mater. 24, 313–321. doi: 10.1016/j.bioactmat.2022.12.026 36632502 PMC9813528

[B48] Livak K. J. Schmittgen T. D. (2001). Analysis of relative gene expression data using real-time quantitative PCR and the 2^-^ΔΔCt method. Methods 25, 402–408. doi: 10.1006/meth.2001.1262 11846609

[B49] Love M. I. Huber W. Anders S. (2014). Moderated estimation of fold change and dispersion for RNA-seq data with DESeq2. Genome Biol. 15, 550. doi: 10.1186/s13059-014-0550-8 25516281 PMC4302049

[B50] Lu L. Qanmber G. Li J. Pu M. Chen G. Li S. . (2021). Identification and characterization of the ERF subfamily B3 group revealed GhERF13.12 improves salt tolerance in upland cotton. Front. Plant Sci. 12, 705883. doi: 10.3389/fpls.2021.705883 34434208 PMC8382128

[B51] Lu S. Wang J. Chitsaz F. Derbyshire M. K. Geer R. C. Gonzales N. R. . (2020). CDD/SPARCLE: the conserved domain database in 2020. Nucleic Acids Res. 48, D265–D268. doi: 10.1093/nar/gkz991 31777944 PMC6943070

[B52] Luo L. Zhang L. Gu R. Ni S. Yu J. Gao Y. . (2025). Genome-wide identification and functional analysis of AP2/ERF gene family in Passiflora edulis Sims. Plants (Basel) 14, 645. doi: 10.3390/plants14050645 40094515 PMC11901831

[B53] Ma Z. Jin Y. M. Wu T. Hu L. Zhang Y. Jiang W. . (2022). OsDREB2B, an AP2/ERF transcription factor, negatively regulates plant height by conferring GA metabolism in rice. Front. Plant Sci. 13, 1007811. doi: 10.3389/fpls.2022.1007811 36388558 PMC9650310

[B54] Park S. Ahn E. Zhang D. Meinhardt L. W. (2025). Comparative genome-wide characterization and evolutionary insights into the AP2/ERF gene family in three Coffea species (C. canephora, C. eugenioides, and C. arabica). BMC Genomics 26, 653. doi: 10.1186/s12864-025-11850-0 40640702 PMC12247349

[B55] Rashid M. Guangyuan H. Guangxiao Y. Hussain J. Xu Y. (2012). AP2/ERF transcription factor in rice: genome-wide canvas and syntenic relationships between monocots and eudicots. Evol. Bioinform. Online 8, 321–355. doi: 10.4137/ebo.s9369 22807623 PMC3396566

[B56] Riaz M. W. Lu J. Shah L. Yang L. Chen C. Mei X. D. . (2021). Expansion and molecular characterization of AP2/ERF gene family in Wheat (Triticum aestivum L.). Front. Genet. 12, 632155. doi: 10.3389/fgene.2021.632155 33868370 PMC8044323

[B57] Salas-Muñoz S. Gómez-Anduro G. Delgado-Sánchez P. Rodríguez-Kessler M. Jiménez-Bremont J. F. (2012). The Opuntia streptacantha OpsHSP18 gene confers salt and osmotic stress tolerance in Arabidopsis thaliana. Int. J. Mol. Sci. 13, 10154–10175. doi: 10.3390/ijms130810154 22949853 PMC3431851

[B58] Seok H. Y. Tran H. T. Lee S. Y. Moon Y. H. (2022). AtERF71/HRE2, an Arabidopsis AP2/ERF transcription factor gene, contains both positive and negative cis-regulatory elements in its promoter region involved in hypoxia and salt stress responses. Int. J. Mol. Sci. 23, 5310. doi: 10.3390/ijms23105310 35628120 PMC9140466

[B59] Shigyo M. Hasebe M. Ito M. (2006). Molecular evolution of the AP2 subfamily. Gene 366, 256–265. doi: 10.1016/j.gene.2005.08.009 16388920

[B60] Shu Y. Liu Y. Zhang J. Song L. Guo C. (2015). Genome-wide analysis of the AP2/ERF superfamily genes and their responses to abiotic stress in Medicago truncatula. Front. Plant Sci. 6, 1247. doi: 10.3389/fpls.2015.01247 26834762 PMC4717309

[B61] Song Y. Gao M. Xu Z. Wang J. Bi M. (2023). “ Temporal and spatial characteristics of soil salinization and its impact on cultivated land productivity in the BOHAI Rim region,” in Water, vol. 15. , 2368.

[B62] Song X. Li Y. Hou X. (2013). Genome-wide analysis of the AP2/ERF transcription factor superfamily in Chinese cabbage (Brassica rapa ssp. pekinensis). BMC Genomics 14, 573. doi: 10.1186/1471-2164-14-573 23972083 PMC3765354

[B63] Song H. Xin J. Yang D. Dong G. Deng X. Liu J. . (2024). NnSUS1 encodes a sucrose synthase involved in sugar accumulation in lotus seed cotyledons. Plant Physiol. Biochem. 210, 108591. doi: 10.1016/j.plaphy.2024.108591 38583314

[B64] Tan Z. W. Lu D. D. Yu Y. L. Li L. Xu L. J. Dong W. . (2025). Genome-wide analysis of the APETALA2/Ethylene-responsive factor gene family in Carthamus tinctorius L. Plant Direct 9, e70032. doi: 10.1002/pld3.70032 39822732 PMC11736709

[B65] Thompson J. D. Higgins D. G. Gibson T. J. (1994). CLUSTAL W: improving the sensitivity of progressive multiple sequence alignment through sequence weighting, position-specific gap penalties and weight matrix choice. Nucleic Acids Res. 22, 4673–4680. doi: 10.1093/nar/22.22.4673 7984417 PMC308517

[B66] Tian Z. He Q. Wang H. Liu Y. Zhang Y. Shao F. . (2015). The potato erf transcription factor StERF3 negatively regulates resistance to phytophthora infestans and salt tolerance in potato. Plant Cell Physiol. 56, 992–1005. doi: 10.1093/pcp/pcv025 25681825

[B67] Todaka D. Nakashima K. Shinozaki K. Yamaguchi-Shinozaki K. (2012). Toward understanding transcriptional regulatory networks in abiotic stress responses and tolerance in rice. Rice (N Y) 5, 6. doi: 10.1186/1939-8433-5-6 24764506 PMC3834508

[B68] Wang L. Ma H. Lin J. (2019). Angiosperm-wide and family-level analyses of AP2/ERF genes reveal differential retention and sequence divergence after whole-genome duplication. Front. Plant Sci. 10, 196. doi: 10.3389/fpls.2019.00196 30863419 PMC6399210

[B69] Wang L. Wang S. Tong R. Wang S. Yao J. Jiao J. . (2022). Overexpression of PgCBF3 and PgCBF7 transcription factors from pomegranate enhances freezing tolerance in Arabidopsis under the promoter activity positively regulated by PgICE1. Int. J. Mol. Sci. 23, 9439. doi: 10.3390/ijms23169439 36012703 PMC9408969

[B70] Wang Y. H. Zhao B. Y. Ye X. Du J. Song J. L. Wang W. J. . (2024). Genome-wide analysis of the AP2/ERF gene family in Pennisetum glaucum and the negative role of PgRAV_01 in drought tolerance. Plant Physiol. Biochem. 216, 109112. doi: 10.1016/j.plaphy.2024.109112 39265240

[B71] Xie Z. Nolan T. M. Jiang H. Yin Y. (2019). AP2/ERF transcription factor regulatory networks in hormone and abiotic stress responses in Arabidopsis. Front. Plant Sci. 10, 228. doi: 10.3389/fpls.2019.00228 30873200 PMC6403161

[B72] Xing H. Jiang Y. Zou Y. Long X. Wu X. Ren Y. . (2021). Genome-wide investigation of the AP2/ERF gene family in ginger: evolution and expression profiling during development and abiotic stresses. BMC Plant Biol. 21, 561. doi: 10.1186/s12870-021-03329-3 34823471 PMC8620233

[B73] Xu Y. Jiang J. Zeng L. Liu H. Jin Q. Zhou P. . (2024). Genome-wide identification and analysis of ERF transcription factors related to abiotic stress responses in Nelumbo nucifera. BMC Plant Biol. 24, 1057. doi: 10.1186/s12870-024-05772-4 39516727 PMC11545801

[B74] Yang H. He S. Feng Q. Liu Z. Xia S. Zhou Q. . (2024). Lotus (Nelumbo nucifera): a multidisciplinary review of its cultural, ecological, and nutraceutical significance. Bioresour. Bioprocess. 11, 18. doi: 10.1145/3275495.3275503 38647851 PMC10991372

[B75] Yang H. Sun Y. Wang H. Zhao T. Xu X. Jiang J. . (2021). Genome-wide identification and functional analysis of the ERF2 gene family in response to disease resistance against Stemphylium lycopersici in tomato. BMC Plant Biol. 21, 72. doi: 10.1186/s12870-021-02848-3 33530947 PMC7856819

[B76] Yu Y. Liu A. Duan X. Wang S. Sun X. Duanmu H. . (2016). GsERF6, an ethylene-responsive factor from Glycine soja, mediates the regulation of plant bicarbonate tolerance in Arabidopsis. Planta 244, 681–698. doi: 10.1007/s00425-016-2532-4 27125386

[B77] Yu G. Wang L. G. Han Y. He Q. Y. (2012). clusterProfiler: an R package for comparing biological themes among gene clusters. OMICS 16, 284–287. doi: 10.1089/omi.2011.0118 22455463 PMC3339379

[B78] Yu Z. Zhang D. Hu S. Liu X. Zeng B. Gao W. . (2022). Genome-wide analysis of the almond AP2/ERF superfamily and its functional prediction during dormancy in response to freezing stress. Biol. (Basel) 11, 1520. doi: 10.3390/biology11101520 36290423 PMC9598233

[B79] Zhang G. Chen M. Li L. Xu Z. Chen X. Guo J. . (2009). Overexpression of the soybean GmERF3 gene, an AP2/ERF type transcription factor for increased tolerances to salt, drought, and diseases in transgenic tobacco. J. Exp. Bot. 60, 3781–3796. doi: 10.3390/ijms23095084 19602544 PMC2736888

[B80] Zhang J. Liao J. Ling Q. Xi Y. Qian Y. (2022). Genome-wide identification and expression profiling analysis of maize AP2/ERF superfamily genes reveal essential roles in abiotic stress tolerance. BMC Genomics 23, 125. doi: 10.1186/s12864-022-08345-7 35151253 PMC8841118

[B81] Zhang Z. Liu H. Sun C. Ma Q. Bu H. Chong K. . (2018). A C2H2 zinc-finger protein OsZFP213 interacts with OsMAPK3 to enhance salt tolerance in rice. J. Plant Physiol. 229, 100–110.1. doi: 10.1016/j.jplph.2018.07.003 30055519

[B82] Zhang H. Wang S. Zhao X. Dong S. Chen J. Sun Y. . (2024). Genome-wide identification and comprehensive analysis of the AP2/ERF gene family in Prunus sibirica under low-temperature stress. BMC Plant Biol. 24, 883. doi: 10.1186/s12870-024-05601-8 39342089 PMC11438396

[B83] Zhao J. Huang M. Liu J. Cai J. He Y. Zhao W. . (2025). Pepper (Capsicum annuum L.) AP2/ERF transcription factor, CaERF2 enhances salt stress tolerance through ROS scavenging. Theor. Appl. Genet. 138, 44. doi: 10.1007/s00122-025-04823-0 39899078

[B84] Zhu Y. Liu X. Gao Y. Li K. Guo W. (2021). Transcriptome-based identification of AP2/ERF family genes and their cold-regulated expression during the dormancy phase transition of Chinese cherry flower buds. Sci. Hortic. 275, 109666. doi: 10.1016/j.scienta.2020.109666 38826717

[B85] Zilu N. I. U. Chunling S. Lei W. Qi T. Chen M. Jiang S. . (2025). Spatial variability of soil salinization and alkalization in the northern plain irrigation area of Yinchuan, Ningxia. JRE 16, 148–158. doi: 10.5814/j.issn.1674-764x.2025.01.014

[B86] Zumajo-Cardona C. Pabón-Mora N. (2016). Evolution of the APETALA2 gene lineage in seed plants. Mol. Biol. Evol. 33, 1818–1832. doi: 10.1093/hesc/9780198815723.003.0006 27030733

