# Supplementary material for: Comprehensive characterization of the genes in AP2/ERF family and their involvement in salt–alkali stress response during Nelumbo nucifera seed germination
Source: Front Plant Sci. 2026 Aug 27;17:1922760. doi: 10.3389/fpls.2026.1922760 (PMC13559889; doi:10.3389/fpls.2026.1922760)
Supplement: Supplementary file 1 [file SupplementaryFile1.docx]

Supplementary Material

## 1.1 Supplementary Figures

**Supplementary Figure 1.** Synteny analysis of AP2/ERF genes between N. nucifera and A. thaliana. Gray lines indicate all syntenic gene pairs between the two species, and red lines highlight the syntenic relationships specifically associated with AP2/ERF genes. Orange represents the chromosomal segments of N. nucifera, and green represents those of A. thaliana.

**Supplementary Figure 2.** Conserved motif analysis of AP2/ERF proteins in *N. nucifera*.A total of 10 distinct conserved motifs were identified among AP2/ERF family proteins using MEME Suite.

**Supplementary Figure 3**. Heatmaps of differentially expressed genes (DEGs) identified in T5 vs. Y5 and T10 vs. Y10 comparisons.(A-B) Heatmap showing expression patterns of DEGs between T5 vs.Y5 and T10 vs. Y10 groups. Red indicates upregulated genes, while blue represents downregulated genes. Gene expression levels were normalized and clustered to reveal expression trends under salt-alkaline stress at different treatment durations.The color scale represents normalized expression levels (log2-transformed values.

**Supplementary Figure 4**. KEGG pathway enrichment of DEGs under salt–alkaline stress.
(A) Enriched KEGG pathways in DEGs between 0 mM (T5) and 150 mM(Y5) treatments after 5 days. Dot size indicates the number of DEGs in each pathway, and color intensity represents the q-value. (B) Enriched KEGG pathways in DEGs between 0 mM (T10) and 150 mM(Y10) treatments after 10 days. Dot size corresponds to the number of DEGs, and dot color reflects the q-value, with darker shades indicating higher significance.

## 1.2  Supplementary Tables

**Supplementary Table 1. Primers were used in this study.**

| **Primer name** | **Primer sequence (5'-3')** | **Primer sequence 5'-3'location** |
| --- | --- | --- |
| AP2-9F | ACACCCGAGGATCGGTGAAG | 565 to 584 |
| AP2-9R | GAGTTTGAGTAACCGCCCAAGA | 759 to 780 |
| ERF23F | GATACGCTGCCGAGATAAGGG | 348 to 368 |
| ERF23R | CTTTAACCGAAGAAGCACCCTC | 611 to 632 |
| ERF15F | TAATGAATTCTCGCCGGAATCT | 149 to 170 |
| ERF15R | CTTCGAACGCCTCTGTAGGACT | 379 to 400 |
| ERF31F | AACTCCCGACGATAAGTCGG | 481 to 500 |
| ERF31R | TGTCAAAGGTGCCAAGCCATA | 679 to 699 |
| ERF34F | GCCAGTTGCCTGAAGCAGTT | 728 to 747 |
| ERF34R | ATCTCTGCTGCCCATTTCCC | 193 to 212 |
| DREB21F | AAGATTCCACCACACCATCG | 61 to 80 |
| DREB21R | TGCCGCTGCTGTCTGGATAT | 361 to 380 |
| ACTIN-F | ATGGCAGATTCCGAGGATATT | From Song et al., |
| ACTIN-R | CTTCAGGAGCTACACGGAGC | From Song et al., |

Note: Primer sequences were designed using Primer5 software. The 5′–3′ genomic locations of the primers were verified by BLAST analysis against the NCBI database based on the Nelumbo nuciferareference genome assembly (GCA_000365185).

**Supplementary Table 2. Homologous gene pairs**

| Gene ID in N. nucifera**.** | Gene ID in *A. thaliana*. |
| --- | --- |
| LOC104600346 | AT1G64380 |
| LOC104600304 | AT2G46310 |
| LOC104600304 | AT4G23750 |
| LOC104600304 | AT4G11140 |
| LOC104600304 | AT5G53290 |
| LOC104608167 | AT1G53170 |
| LOC104608167 | AT1G28360 |
| LOC104608167 | AT3G15210 |
| LOC104586457 | AT2G20880 |
| LOC104609267 | AT1G53910 |
| LOC104609294 | AT1G19210 |
| LOC104609334 | AT1G72570 |
| LOC104609267 | AT3G14230 |
| LOC104600117 | AT1G78080 |
| LOC104600117 | AT1G22190 |
| LOC104600117 | AT1G36060 |
| LOC104600117 | AT4G39780 |
| LOC104599945 | AT5G64750 |
| LOC104602399 | AT2G44940 |
| LOC104602399 | AT3G60490 |
| LOC104606213 | AT2G47520 |
| LOC104606213 | AT3G16770 |
| LOC104603385 | AT1G80580 |
| LOC104593412 | AT2G46310 |
| LOC104593412 | AT4G23750 |
| LOC104593412 | AT5G53290 |
| LOC104603172 | AT5G57390 |
| LOC104611932 | AT5G65510 |
| LOC104611932 | AT5G10510 |
| LOC104593077 | AT1G24590 |
| LOC104593077 | AT1G12980 |
| LOC104592717 | AT1G28160 |
| LOC104592746 | AT2G33710 |
| LOC104592746 | AT5G61890 |
| LOC104592717 | AT5G13910 |
| LOC104592746 | AT5G13330 |
| LOC104600857 | AT2G31230 |
| LOC104600857 | AT3G23220 |
| LOC104600857 | AT5G43410 |
| LOC104612693 | AT1G12630 |
| LOC104612693 | AT5G52020 |
| LOC104603036 | AT1G25560 |
| LOC104602974 | AT1G68550 |
| LOC104603036 | AT1G68840 |
| LOC104603036 | AT1G13260 |
| LOC104603036 | AT3G25730 |
| LOC104602974 | AT3G25890 |
| LOC104600466 | AT1G72360. |
| LOC104600466 | AT1G53910 |
| LOC104600498 | AT1G19210 |
| LOC104600466 | AT3G14230 |
| LOC104592437 | AT5G25390 |
| LOC104601351 | AT1G13260 |
| LOC104601351 | AT1G68840 |
| LOC104601351 | AT1G25560 |
| LOC104601805 | AT1G68550 |
| LOC104601351 | AT3G25730 |
| LOC104601805 | AT3G25890 |
| LOC104599450 | AT1G28360 |
| LOC104599450 | AT3G15210 |
| LOC104599450 | AT5G44210 |
| LOC104608486 | AT1G77640 |
| LOC104599345 | AT5G60120 |
| LOC104595388 | AT1G15360 |
| LOC104595123 | AT3G16280 |
| LOC104595123 | AT4G32800 |
| LOC104609654 | AT1G77640 |
| LOC104609654 | AT1G21910 |
| LOC104604040 | AT1G01250 |
| LOC104598888 | AT2G47520 |
| LOC104598888 | AT3G16770 |
| LOC104610490 | AT2G44840 |
| LOC104610488 | AT4G17490 |
| LOC104610490 | AT4G17500 |
| LOC104610488 | AT5G61600 |
| LOC104610490 | AT5G47220 |
| LOC104610488 | AT5G47230 |
| LOC104610488 | AT5G07580 |
| LOC104601725 | AT2G23340 |
| LOC104601725 | AT4G36900 |
| LOC104601725 | AT5G67190 |
| LOC104598425 | AT2G44840 |
| LOC104598459 | AT2G44940 |
| LOC104598459 | AT2G35700 |
| LOC104598459 | AT3G60490 |
| LOC104598426 | AT4G17490 |
| LOC104598425 | AT4G17500 |
| LOC104598425 | AT5G47220 |
| LOC104598426 | AT5G07580 |
| LOC104598426 | AT5G61600 |
| LOC104591971 | AT1G12980 |
| LOC104591971 | AT1G24590 |
| LOC104592013 | AT2G33710 |
| LOC104592013 | AT5G13330 |
| LOC104592013 | AT5G07310 |
| LOC104592013 | AT5G61890 |
| LOC104591899 | AT5G13910 |
| LOC104591308 | AT2G36450 |
| LOC104591238 | AT4G37750 |
| LOC104586938 | AT2G36450 |
| LOC104605948 | AT1G78080 |
| LOC104605948 | AT1G22190 |
| LOC104605948 | AT1G36060 |
| LOC104605948 | AT2G22200 |
| LOC104605948 | AT4G39780 |
| LOC104610030 | AT2G31230 |
| LOC104610031 | AT3G23230 |
| LOC104610030 | AT3G23240 |
| LOC104610031 | AT5G43410 |
| LOC104606875 | AT1G46768 |
| LOC104606875 | AT2G23340 |
| LOC104606875 | AT3G50260 |
| LOC104609075 | AT4G37750 |
| LOC104606875 | AT4G36900 |
| LOC104606875 | AT5G67190 |
| LOC104596183 | AT1G12630 |
| LOC104596183 | AT5G52020 |

Note: Gene duplication events in *N. nucifera* were detected using MCScanX based on BLASTp results (E-value < 1 × 10⁻⁵). Collinear blocks were defined with a maximum gap of 25 genes and a minimum of five gene pairs per block. In addition, syntenic gene pairs between *N. nucifera* and *A. thaliana* were identified and visualized using TBtools. The table lists duplicated gene pairs within *N. nucifera* and orthologous collinear pairs between the two species.

**Supplementary Table 3. Sequencing data statistics in *N. nucifera***

| **Sample ID** | **Raw Data Reads** | **Clean Data Reads** | **Raw Data Base** | **Clean Data Base** | **Q20** | **Q30** |
| --- | --- | --- | --- | --- | --- | --- |
| T5-1 | 36,399,634 | 35,828,573 | 10,919,890,200 | 10,466,149,061 | 98.66 | 95.65 |
| T5-2 | 35,162,531 | 34,300,210 | 10,548,759,300 | 9,975,676,251 | 98.66 | 95.74 |
| T5-3 | 41,600,751 | 40,243,070 | 12,480,225,300 | 11,786,986,986 | 98.45 | 953 |
| T5 average | 37,720,972 | 36,790,617 | 11,316,291,600 | 10,742,937,432 | 98.59 | 95.51 |
| Y5-1 | 44,797,056 | 43,454,563 | 13,439,116,800 | 12,707,503,980 | 98.46 | 956 |
| Y5-2 | 28,997,737 | 27,697,872 | 8,699,321,100 | 8,190,925,679 | 98.6 | 95.48 |
| Y5-3 | 38,936,470 | 37,783,844 | 11,680,941,000 | 10,932,298,245 | 98.64 | 95.65 |
| Y5 average | 37,577,087 | 36,312,093 | 11,273,126,300 | 10,610,242,634 | 98.56 | 95.43 |
| T10-1 | 34,956,103 | 34,304,091 | 10,486,830,900 | 10,168,252,910 | 98.44 | 95.04 |
| T10-2 | 37,605,561 | 36,692,736 | 11,281,668,300 | 10,851,930,843 | 98.48 | 95.24 |
| T10-3 | 42,298,232 | 41,316,840 | 12,689,469,600 | 12,116,199,026 | 98.5 | 95.25 |
| T10 average | 38,286,632 | 37,437,889 | 11,485,989,600 | 11,045,460,926 | 98.47 | 958 |
| Y10-1 | 33,051,851 | 31,634,357 | 9,915,555,300 | 9,346,841,537 | 98.62 | 95.52 |
| Y10-2 | 30,618,315 | 29,420,437 | 9,185,494,500 | 8,733,589,627 | 98.4 | 94.93 |
| Y10-3 | 41,639,258 | 40,489,310 | 12,491,777,400 | 11,905,503,442 | 98.74 | 95.93 |
| Y10 average | 35,103,141 | 33,848,034 | 10,530,942,400 | 9,995,311,535 | 98.58 | 95.46 |

Note: The table presents sequencing quality metrics for each sample, including total raw reads, clean reads after quality filtering, raw and clean base counts, and quality scores (Q20 and Q30). Clean reads were obtained after removing adaptor sequences, low-quality reads, and ambiguous bases. Q20 and Q30 represent the percentages of bases with Phred quality scores ≥20 and ≥30, respectively, indicating sequencing accuracy and overall data reliability.
